# Efficacy of emergency maternal MVA-ZIKV vaccination in a rapid challenge model of lethal Zika infection

**DOI:** 10.1038/s41541-025-01094-0

**Published:** 2025-03-05

**Authors:** Asisa Volz, Sabrina Clever, Alina Tscherne, Astrid Freudenstein, Sylvia Jany, Jan H. Schwarz, Leonard Limpinsel, William G. Valiant, Georgia Kalodimou, Gerd Sutter, Joseph J. Mattapallil

**Affiliations:** 1 Institute of Virology, University of Veterinary Medicine Hannover, Hannover, Germany; 2 Division of Virology, Department of Veterinary Sciences, LMU Munich, Munich, Germany; 3 German Center for Infection Research, Partner Site Hannover-Braunschweig, Braunschweig, Germany; 4 German Center for Infection Research, Partner Site Munich, Munich, Germany; 5 Dept. of Microbiology & Immunology, Uniformed Services University, Bethesda, MD USA

**Keywords:** Immunology, Microbiology

## Abstract

Zika virus (ZIKV) outbreak of 2015 was associated with microcephaly and congenital birth defects in children born to pregnant women infected with ZIKV. Using the highly susceptible Type I Interferon Receptor-deficient mouse-model, we demonstrate that a single emergency vaccination with a non-replicating MVA-ZIKV vaccine, when administered as early as 2-days before challenge fully protected non-pregnant and pregnant mice and fetuses against lethal ZIKV-infection. Early protection was associated with the rapid emergence of ZIKV-specific CD8+ T cell responses; depletion of CD8+ T cells resulted in the loss of protection supporting a critical role for CD8+ T cells in the early protective efficacy of MVA-ZIKV. Neutralizing antibody responses were induced later than the CD8+ T cell responses, suggesting that it may play a role in later stages of infection. Our results suggest that MVA-ZIKV induces potent anamnestic cellular immunity early after infection, contributing to its protective efficacy against rapid ZIKV challenge.

## Introduction

Zika virus (ZIKV) causes Zika fever, a mild disease with fever, joint pain, headache, and a maculopapular rash. However, in pregnant women, ZIKV infection has been associated with Congenital Zika Syndrome (CZS) characterized by abortions, microcephaly and other brain malformations in the fetus. Interestingly, CZS is more apparent in pregnant women who are infected during the first trimester of pregnancy^1^ narrowing the window of opportunity to protect the fetus. There is limited information regarding the protective efficacy of ZIKV vaccines during early pregnancy when administered immediately before ZIKV exposure^2,3^. Numerous ZIKV-prME-based vaccines have been tested in pre-clinical and advanced clinical trials with potent efficacy against ZIKV infection^2,4–10^.

Most ZIKV immunogenicity studies have relied on testing efficacy after confirming the induction of potent ZIKV-specific neutralizing antibody (nAb) and T cell responses followed by ZIKV challenge weeks after vaccination. As such, little information is available regarding the efficacy of ZIKV vaccines in emergencies such as travel to ZIKV endemic areas or an outbreak. Pregnant women remain the most vulnerable to ZIKV infection, given its association with CZS, especially in ZIKV endemic areas.

Testing the efficacy of a vaccine to protect vulnerable populations remains a priority. We tested this hypothesis using a rapid challenge type I interferon (IFN) receptor-deficient (IFNAR-/-) pregnant mouse lethal infection model using a non-replicating MVA-ZIKV vaccine.

MVA-based vaccines provide a versatile, non-replicating viral vector vaccine platform with a well-established safety profile^11–13^ as it readily infects but does not replicate in human cells, allowing for unimpaired cytoplasmic expression of proteins of interest^11,14–16^. Currently, non-replicating MVA-based vaccines have been licensed for human use against mpox and smallpox (https://www.fda.gov/vaccines-blood-biologics/jynneos, https://www.ema.europa.eu/en/medicines/human/EPAR/imvanex) and Ebola (https://www.ema.europa.eu/en/medicines/human/EPAR/mvabea)^17^, and found to be safe in numerous pre-clinical and clinical trials, including human subjects with immuno-deficiencies^18,19^, atopic dermatitis^20^ and stem-cell transplant recipients^21^, and in elderly individuals^22^.

Perez et al.^14^ tested the efficacy of an MVA-ZIKV-prME (MVA-ZIKV) vaccine using immunodeficient mouse models^14^. Mice were vaccinated intraperitoneally (IP) with either one or two doses of the MVA-ZIKV vaccine followed by ZIKV challenge 4 weeks later (10 days after the last immunization). At 4 weeks post-vaccination, both nAb and T cell responses were detectable and correlated with control of ZIKV infection. The kinetics of either nAb or T cell responses since the time of first immunization was not determined. As such, it is not clear if immune responses were induced immediately after vaccination. We sought to determine if the MVA-ZIKV vaccine could protect from ZIKV infection if vaccinated immediately before the challenge.

Our results demonstrated that a single dose of the MVA-ZIKV vaccine administered intramuscularly (IM) in the early 2-days before the highly pathogenic ZIKV challenge fully protected IFNAR-/- non-pregnant and pregnant mice against lethal infection and prevented in-utero transmission of ZIKV. Protection correlated with the rapid emergence of ZIKV-E-protein-specific anamnestic CD8+ T cell responses as early as 2 days after the challenge. In contrast, binding antibody (bAb) and nAb responses were detected much later than the emergence of protective CD8+ T cell responses as reported in earlier studies^14^, suggesting that the rapid induction of potent CD8+ T cell responses rapidly cleared ZIKV infection and protected against adverse outcomes during pregnancy.

## Results

### Design and generation of a recombinant MVA-ZIKV vaccine

**Fig. 1:**
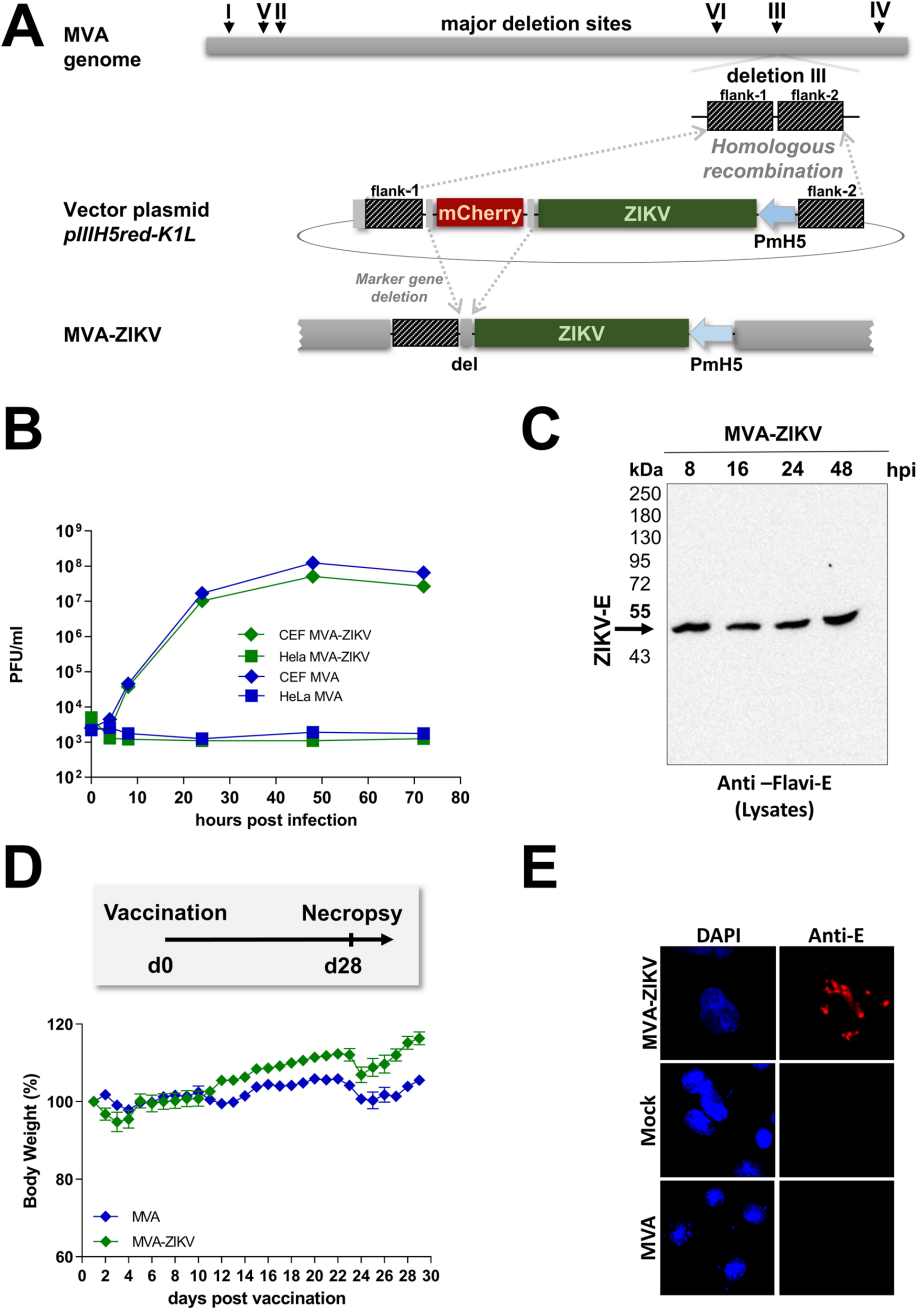
Design and in vitro characterization of MVA-ZIKV vaccine. **A** Schematic diagram of the MVA genome with the major deletion sites I–VI. The site of deletion III was targeted for the insertion of the gene sequence encoding the -prME protein of Zika virus isolate Yap 2007 (ZIKV-prME) under transcriptional control of the vaccinia virus promoter PmH5 within the MVA vector plasmid pIIIH5red-K1L. Insertion occurred via homologous recombination between MVA DNA sequences (flank-1 and flank-2) adjacent to deletion site III in the MVA genome to generate MVA-prME (MVA-ZIKV). **B** Multiple-step growth analysis of recombinant MVA-ZIKV. Cells were infected at an MOI of 0.05 with MVA-ZIKV and empty MVA and collected at the indicated time-points. Titration was performed on CEF cells and PFU were determined. **C** Western blot analysis of ZIKV-E in lysates of MVA-ZIKV-infected cells using a monoclonal antibody against the ZIKV-E protein. **D** In situ immunofluorescence of ZIKV-E protein in MVA, mock, or MVA-ZIKV-infected Vero cells (MOI of 0.5) 16 h post-infection. Cells were probed with a monoclonal antibody against the ZIKV-E protein. Goat anti-mouse antibody for E-specific fluorescent staining (red). Cell nuclei were counterstained with DAPI (blue). **E** Body weights of IFNAR-/- mice that were vaccinated with either MVA-ZIKV or empty MVA-vector at 28 days. The error bars indicate for mean ± standard error of the mean (SEM).

Modified Vaccinia Ankara vector was used to construct the MVA-based ZIKV-prME vaccine (MVA-ZIKV). Gene sequences encoding the prME-protein from Amino acids 126-790 of ZIKV Yap 2007 isolate (GenBank accession no. EU545988.1YYYY, Fig. S1) was inserted into the MVA transfer plasmid pIIIH5red-K1L. The heterologous gene was placed under the transcriptional control of the enhanced synthetic vaccinia virus early/late promoter PmH5 (Fig. 1A). Clonal recombinant MVA viruses expressing ZIKV-prME were isolated by repetitive plaque purification using transient coproduction of the fluorescent marker protein mCherry to screen for red fluorescent cell foci.

Insertion was confirmed by PCR-analysis of viral genomic DNA using specific oligonucleotide primers targeting sequences adjacent to the MVA deletion site III (Fig. S2A). Genetic integrity of MVA-ZIKV was confirmed by using specific oligonucleotide primers targeting the respective ZIKV-gene sequence (Fig. S2B). In addition, genetic identity and stability were verified using oligonucleotide primers specific to the six major deletion sites within the MVA genome (Fig. S2C) and the C7L gene locus (Fig. S2D). The replication capacity of MVA-ZIKV constructs was tested in chicken embryo fibroblast cells (CEF) and human HeLa cells to verify the conservation of the well-established MVA phenotype as a replication-deficient vector. As expected, MVA-ZIKV replicated efficiently in CEF, but not in the human HeLa cell line (Fig. 1B) confirming that the MVA-ZIKV did not replicate in human cells as has been reported previously^11,14–16^.

To confirm the expression of prME protein, CEF cells were infected with the MVA-ZIKV vaccine construct at a multiplicity of infection (MOI) of 0.5 PFU. Total cell lysates were collected and analyzed over 48 h by Western blots using a pan-flavivirus-E-specific antibody. In line with earlier studies^23–25^, we observed a single protein band with a molecular mass of ∼48 kDa corresponding to the E protein in the cell lysates as early as 8 h after infection that remained stable at high levels over 48 h (Fig. 1C). The intracellular expression of the ZIKV-prME -protein was confirmed by immunofluorescence staining (Fig. 1D).

To determine if MVA vaccination would lead to an adverse outcome in immune-deficient mice, we vaccinated 6–8 weeks old IFNAR-/- mice intramuscularly with 10^8^ PFU (plaque-forming units) of the MVA-ZIKV vaccine and compared them to controls. Mice were monitored daily for a period of 28-days for changes in body weight (BW). No change in BW (Fig.1E) was observed in either MVA-vaccinated IFNAR-/- mice or PBS control mice that was in line with earlier studies^14^.

### Single-shot MVA-ZIKV vaccination induces ZIKV-specific T cells and ZIKV-specific antibodies in C57BL/6 mice and IFNAR-/- mice

**Fig. 2:**
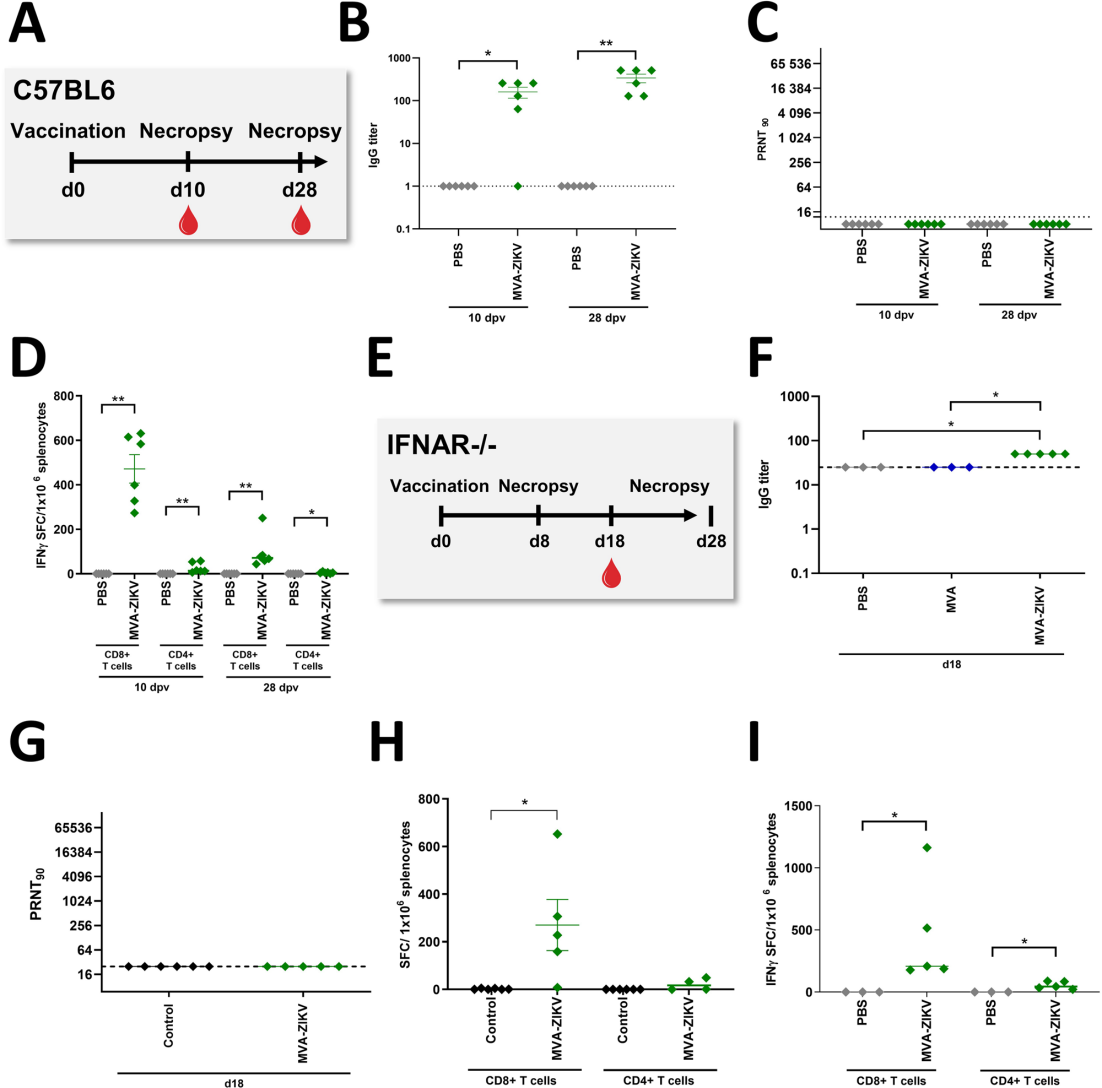
Immunogenicity of MVA-ZIKV vaccination in C57BL/6 mice or IFNAR-/- mice. ZIKV-specific immune responses induced after MVA-ZIKV vaccination in C57BL/6 mice (**A**–**D**) or IFNAR-/- mice (E-I). **A** Vaccination Schedule in C57BL/6 mice. Sera from MVA-ZIKV vaccinated C57BL/6 mice (*n* = 6) and PBS-mock vaccinated C57BL/6 mice (*n* = 6) were prepared at 10 or 28 dpv and analyzed for (**B**) humoral immune responses by ZIKV-E-ELISA and (**C**) for ZIKV-neutralizing antibodies by PRNT_90_. **D** Splenocytes from MVA-ZIKV-vaccinated mice (*n* = 6) and PBS-mock-vaccinated mice (*n* = 6) were prepared at 10 dpv or at 28 dpv and analyzed for IFN-γ SFC after stimulation with E_294-302_ peptide (CD8+ T cells) or E_646-664_ peptide (CD4+ T cells) measured by ELISPOT assay. **E** Vaccination Schedule in IFNAR-/- mice including MVA-ZIKV-vaccinated IFNAR-/- mice (*n* = 5) and MVA-vaccinated mice (*n* = 3) and PBS-mock-vaccinated mice (*n* = 3) were both combined as control group. **F**, **G** Sera from MVA-ZIKV vaccinated IFNAR-/- mice, empty MVA-vector vaccinated mice, and PBS-mock vaccinated mice (combined as control group) were prepared at 18 dpv analyzed. **E**, **F** for humoral immune responses by ZIKV-E-ELISA or (**G**) for ZIKV-neutralizing antibodies by PRNT_90_. Dotted lines mark the limit of detection. **H** Splenocytes from MVA-ZIKV-vaccinated IFNAR-/- mice (*n* = 5), MVA-vaccinated mice (*n* = 3) and PBS-mock-vaccinated mice (*n* = 3, both combined as control group) were prepared at 8 dpv and analyzed for IFN-γ SFC after stimulation with E_294-302_ peptide (CD8+ T cells) or E_646-664_ peptide (CD4+ T cells) measured by ELISPOT assay. **I** Splenocytes from MVA-ZIKV vaccinated IFNAR-/- mice (*n* = 5) and PBS-mock-vaccinated mice (*n* = 3) were prepared at 28 dpv and analyzed for IFN-γ SFC after stimulation with E_294-302_ peptide (CD8+ T cells) or E_646-664_ peptide (CD4+ T cells) measured by ELISPOT assay. Each dot indicates for a single mouse. The horizontal lines and associated error bars indicate for mean ± standard error of the mean (SEM). Differences between the groups were analyzed by two-tailed Mann-Whitney Test (**B**–**D**, **I**) and Kruskal-Wallis Test (**F**–**H**). Asterisks represent statistically significant differences between two groups: * *p* < 0.05, ** *p* < 0.01.

To determine immunogenicity of the MVA-ZIKV vaccine in vivo, adult immunocompetent wild-type (WT) C57BL/6 mice were vaccinated intramuscularly (IM) with a single dose of 10^8^ PFU MVA-ZIKV vaccine (Fig. 2A). Additionally, we tested the immunogenicity of MVA-ZIKV vaccination at a single dose of 10^8^ PFU using the IFNAR-/- model (Fig. 2E). ZIKV-binding antibodies (bAb) and ZIKV-neutralizing antibodies (nAb) along with T cell responses were examined at 10- and 28-days post-vaccination (dpv) in WT C57BL/6 mice. In the IFNAR-/- mice, we analyzed the humoral immune responses at 18 dpv and the T cell responses at 8 and 28 dpv. Antibody responses were detectable in both WT C57BL/6 and IFNAR-/- mice (Fig. 2B, C, F, G).

ZIKV-E-protein-specific CD4+ and CD8+ T cell responses were determined using IFN-γ ELISPOT assay. Freshly isolated splenocytes from immunized and control mice were stimulated with either a CD8+ T cell ZIKV-E-peptide (ZIKV-E_294-302_) or CD4+ T cell ZIKV-E-peptide (ZIKV-E_646-662_) that were shown to be immunodominant in earlier studies^26^. Significant levels of E_294-302_ specific CD8+ T cells were detectable in both WT C57BL/6 (471 Spot Forming Counts (SFC)/million splenocytes, Fig. 2D, day 10 p.v. and day 28 p.v.) and IFNAR-/- mice (~270 SFC, Fig. 2H at day 8 p.v., and Fig. 2I ~450 SFC at day 28 p.v.) as compared to the control groups. Unlike CD8 T cell responses, low levels of CD4 T cell responses were detected in both groups of mice. Additionally, E_294-302_-specific CD8+ T cells (~1560 SFC/million splenocytes) and E_646-662_-specific CD4+ T cells (mean of 75 SFC/million splenocytes) were readily detectable at 6 months post-vaccination suggesting that MVA-ZIKV vaccination induced durable T cell responses (Fig. S3).

### Single-shot MVA-ZIKV vaccination protects IFNAR-/- mice against lethal ZIKV challenge 28-days later

**Fig. 3:**
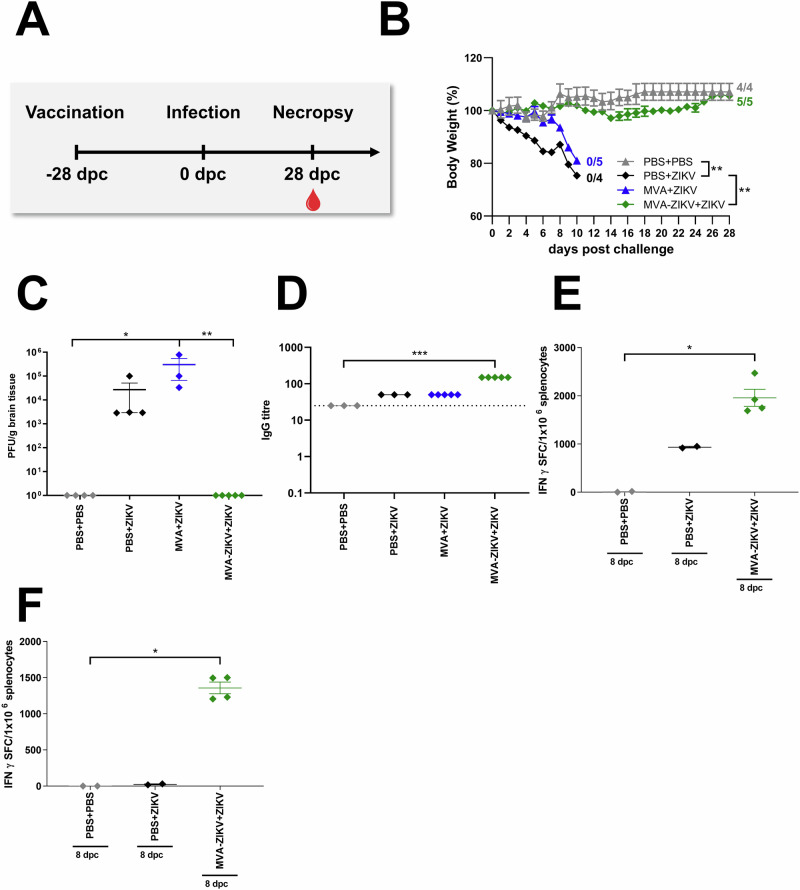
Vaccination with MVA-ZIKV protects against lethal ZIKV challenge at day 28 post vaccination. IFNAR-/- mice were challenged with ZIKV 28-days after intramuscular vaccination with MVA-ZIKV (10^8^ PFU, *n* = 5). Empty MVA (*n* = 4) or PBS-vaccinated mice (mock-vaccinated animals, *n* = 3) were used as vaccination controls. PBS-vaccinated and PBS-challenged mice (mock-mock animals, *n* = 4) were used as infection controls. Body weight changes, clinical scores, viral loads, and immunogenicity were determined. **A** Vaccination-challenge schedule. **B** Body weight change was monitored daily. **C** Brains were taken at the time point of death and analyzed for the amounts of infectious ZIKV PFU/gram tissue. **D** Sera were prepared at the day of death (PBS + ZIKV, MVA + ZIKV: 10-11 dpc, PBS + PBS, MVA-ZIKV + ZIKV: 28 dpc) and analyzed for humoral immune responses by ZIKV-E-ELISA. Dotted lines mark the limit of detection. Splenocytes from mice that were vaccinated at day 0 and challenged with ZIKV at 28 dpv were prepared 8-days after the ZIKV-challenge (day-36 post initial vaccination) and analyzed for IFN-γ SFC after stimulation with (**E**) E_294-302_ peptide (CD8+ T cells) or (**F**) E_646-664_ peptide (CD4+ T cells) measured by ELISPOT assay. Each dot indicates for a single mouse (**C**–**F**). The horizontal lines and associated error bars indicate for mean ± standard error of the mean (SEM). Differences between the groups were analyzed determining the area under the curve (AUC) prior to analysis by Kruskal-Wallis Test (**B**) or single analysis by Kruskal-Wallis Test (**C**–**F**). Asterisks represent statistically significant differences between two groups: ns non-significant, * *p* < 0.05, ** *p* < 0.01, *** *p* < 0.001.

To assess if the MVA-ZIKV vaccine was as efficacious against ZIKV as reported by Perez et al.^14^, adult IFNAR-/- mice were immunized with a single dose of 10^8^ PFU of MVA-ZIKV by the IM route, and 28-days later, challenged subcutaneously (SC) with ZIKV (10^3^ PFU, Fig. 3A) and compared to control IFNAR-/- mice (PBS followed by PBS 28-days later) or PBS + ZIKV (PBS followed by ZIKV infection 28-days later) or MVA + ZIKV (empty MVA vector followed by ZIKV infection 28-days later).

No change in either BW or clinical score was observed in PBS-PBS control mice (Fig. 3B, Fig. S5A). In contrast, PBS + ZIKV and MVA + ZIKV control mice developed severe disease starting at 6 days post-challenge (dpc) that was characterized by a substantial loss of BW, and severe neurologic symptoms, including partial or complete paralysis of one or both hind limbs, reduced activity, and scruffy fur (Fig. 3B, Fig. S5A). All PBS + ZIKV and MVA + ZIKV control mice died due to ZIKV infection within 10–11 dpc, or were euthanized due to a significant loss of BW ranging from 20–25%. In contrast, mice vaccinated with the MVA-ZIKV vaccine experienced a minimal loss of BW (<5%) and were fully protected from clinical disease and death until the end of the experiment at 28 dpc (Fig. 3B, Fig. S5A).

At the time of death, high levels of ZIKV were detected in the brains of both PBS + ZIKV (~2,7 × 10^4^ PFU/gram) and MVA + ZIKV (~3 × 10^5^ PFU/gram, Fig. 3C) control mice whereas no ZIKV was detectable in the brains of MVA-ZIKV vaccinated mice (Fig. 3C), suggesting that vaccination fully protected immune-deficient IFNAR-/- mice from lethal disease. As reported by Perez et al., high titers of ZIKV-specific bAb and nAb responses were detectable in the MVA-ZIKV vaccinated mice compared to control animals (Fig. 3E, Fig. S4).

To determine if ZIKV infection was associated with an anamnestic CD8+ T cell response, we examined ZIKV-E-specific T cells in MVA-ZIKV vaccinated IFNAR-/- mice at day 8 post-infection with ZIKV and compared them to control mice (PBS + PBS, PBS + ZIKV). We observed significant levels of E_294-302_-specific CD8+ T cells (~2000 (SFC)/million splenocytes) and E_646-662_-specific CD4+ T cells (~2000 SFC/million splenocytes) at 8 dpc in MVA-ZIKV vaccinated mice as compared to PBS-control mice (Fig. 3F). These responses were significantly higher than those induced before the challenge (Fig. 2G) with E_294-302_-specific CD8+ T cells (~300 SFC/million splenocytes) and E_646-662_-specific CD4+ T cells (<50 SFC/million splenocytes) suggesting that ZIKV infection was associated with a significant anamnestic increase in MVA-ZIKV vaccine induced CD8+ and CD4+ T cell responses.

### Rapid protective efficacy of a single-shot MVA-ZIKV vaccination in IFNAR-/- mice against lethal ZIKV challenge infection

**Fig. 4:**
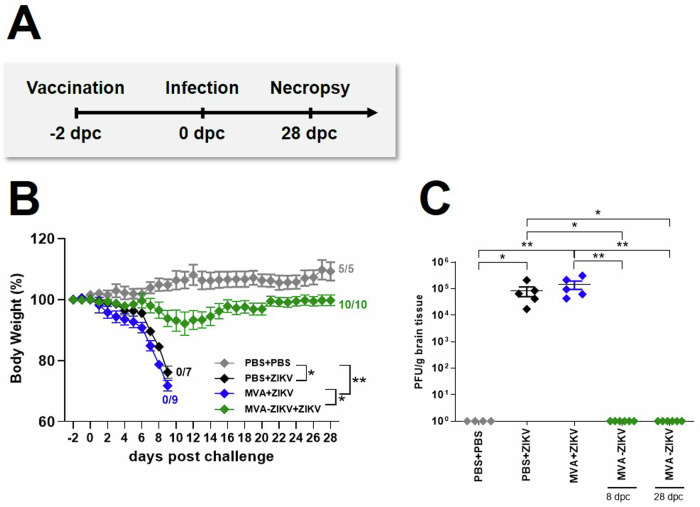
Vaccination with MVA-ZIKV protects against lethal ZIKV challenge at day 2 post vaccination. IFNAR-/- mice were challenged with ZIKV 2-days after intramuscular vaccination with MVA-ZIKV (10^8^ PFU). Body weight changes, clinical scores, viral loads, and immunogenicity were determined. **A** Vaccination-challenge schedule. **B** Body weight change was monitored daily in MVA-ZIKV (*n* = 10) vaccinated mice and compared to empty MVA (*n* = 9) or PBS-vaccinated mice (*n* = 7) that were used as vaccination controls. PBS-vaccinated and PBS-challenged mice (*n* = 5) were used as infection controls. **C** Brains from PBS vaccinated and PBS challenged (*n* = 4), PBS vaccinated and ZIKV challenged (*n* = 5), empty MVA vaccinated and ZIKV challenged (*n* = 5), MVA-ZIKV vaccinated and ZIKV challenged at 8 dpc (*n* = 6) and MVA-ZIKV vaccinated and ZIKV challenged at 28 dpc (*n* = 6) were taken at the time point of death analyzed for the amounts of infectious ZIKV PFU/gram tissue. Each dot indicates for a single mouse. **C**. The horizontal lines and associated error bars indicate for mean ± standard error of the mean (SEM). Differences between the groups were analyzed determining the area under the curve (AUC) prior to analysis by Kruskal-Wallis Test (**B**) or single analysis by Kruskal-Wallis Test (**C**). Asterisks represent statistically significant differences between two groups: * *p* < 0.05, ** *p* < 0.01.

To evaluate if the MVA-ZIKV vaccine would be efficacious in a rapid challenge model, we immunized adult IFNAR-/- mice IM with 10^8^ PFU of MVA-ZIKV and challenged them 2-days later SC with 10^3^ PFU ZIKV. No change in either BW or clinical score was observed in PBS-PBS control mice (Fig. 4B, Fig. S5B). In contrast, PBS + ZIKV and MVA + ZIKV control mice developed severe disease starting at 6 dpc that was characterized by substantial loss of BW, and severe neurologic symptoms, including partial or complete paralysis of one or both hind limbs, reduced activity, and scruffy fur (Fig. 4B, Fig. S5B). All PBS- and MVA-control treated mice died due to ZIKV-infection within 9 dpc, or were euthanized due to a significant loss of BW ranging from 20–25%. (Fig. 4B). In contrast, MVA-ZIKV vaccinated mice experienced a moderate loss of BW (~11%) up to 11 dpc but were fully protected from clinical disease and death (Fig. 4B, Fig. S5B). High levels of ZIKV were detected in the brains of both PBS + ZIKV (~8.3 ×10^4^ PFU) and MVA + ZIKV (~1.4 × 10^5^ PFU, Fig. 4C) control mice at time of death with no detectable virus at the time of euthanasia in the MVA-ZIKV vaccinated mice (28 dpc, Fig. 4C). We did detect low levels of ZIKV in the blood at day 4 pc (~3–4 logs PFU equivalent/ml) but not in the brain 6 dpc. Taken together, these results suggest that the MVA-ZIKV vaccine-induced immune responses were effective in preventing lethal disease in a rapid challenge model of ZIKV infection.

### Rapid protective efficacy of a single-shot vaccination with MVA-ZIKV is associated with delayed induction of neutralizing antibody responses

**Fig. 5:**
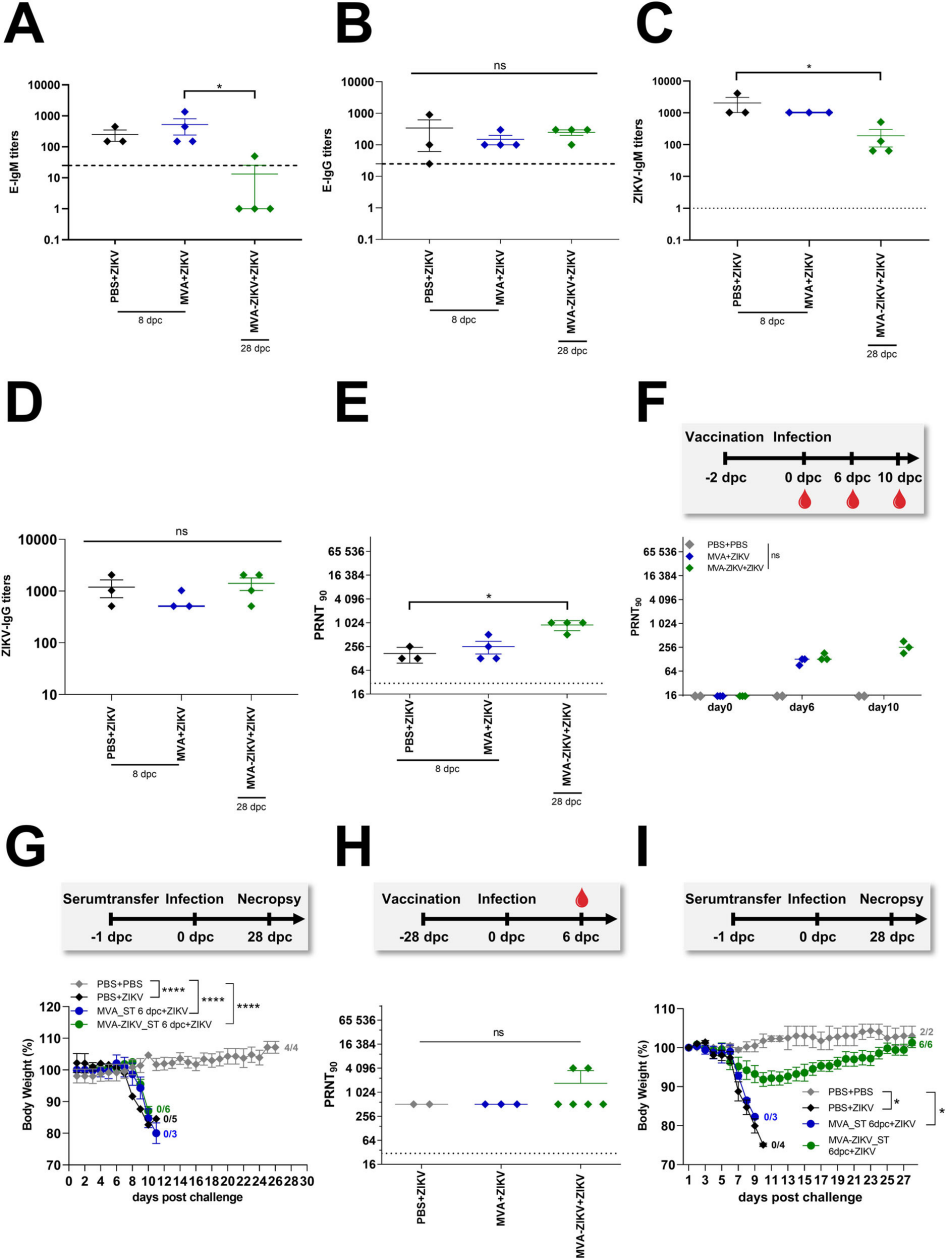
Evaluation of the contribution of antibodies for rapid MVA-ZIKV protection. IFNAR-/- mice were vaccinated with 1 × 10^8^ PFU MVA-ZIKV, empty MVA-vector control and PBS-mock. Mice were challenged 2-days later with ZIKV SC (**A**–**G**). Sera from MVA-ZIKV-vaccinated mice (*n* = 4), MVA-vaccinated mice (*n* = 4) and PBS-mock-vaccinated mice (*n* = 3) were prepared 8 dpc (PBS, MVA) or 28 dpc (MVA-ZIKV) and analyzed for humoral immune responses by ZIKV-E-ELISA (**A**, **B**), ZIKV-ELISA (**C**, **D**) and plaque reduction assay (PRNT90) (**E**). **A** ZIKV-E-specific IgM titers by ELISA and **B** ZIKV-E-specific IgG titers. **C** ZIKV-specific IgM titers and (**D**) ZIKV-specific IgG titers. Dotted lines mark the limit of detection. **E** ZIKV neutralizing antibodies by PRNT_90_ in sera from mice that were vaccinated at day 0 and challenged with ZIKV at 2 dpv. Sera were collected at 8 dpc and 28 dpc. **F** Study design: Preparation of sera for passive transfer: ZIKV neutralizing antibodies in sera from mice that were vaccinated at day-2 and challenged with ZIKV at day 0. Sera were collected at 0, 6 and 10 dpc. **G** Protective capacity of passively transferred serum, as prepared on 6 dpc and analyzed in (**F**) was tested against a lethal ZIKV challenge infection 24 h after serum-transfer (ST) at 6 dpc (8 dpv). Body weight change was monitored daily. **H** Study design: Preparation of sera for passive transfer using the 28-days vaccination schedule: ZIKV neutralizing antibodies in sera from mice that were vaccinated at day-0 and challenged with ZIKV at 28 dpv and sera were collected at 6 dpc (34 dpv) (**I**) Protective capacity of passively transferred serum prepared on 6 dpc and analyzed in (**H**). Body weight change was monitored daily. Each dot indicates a single mouse (**A**–**F**, **H**). The horizontal lines and associated error bars indicate for mean ± standard error of the mean (SEM). Differences between groups were analyzed by Kruskal-Wallis Test (**A**–**F**, **H**) or by determining the area under the curve (AUC) prior to analysis by one-way ANOVA (**G**) or prior to analysis by Kruskal-Wallis Test (**I**). Asterisks represent statistically significant differences between two groups: ns non-significant, * *p* < 0.05, **** *p* < 0.0001.

Antibody responses play a critical role in protection. To assess if the protective efficacy of MVA-ZIKV vaccination in the rapid challenge model was mediated by antibody responses, we vaccinated adult IFNAR-/- mice at day-2 and then challenged them with ZIKV 2 days later, and compared them to control mice (PBS + PBS and MVA + ZIKV). Higher titers of ZIKV-E-protein-specific or whole live ZIKV specific IgM and IgG titerss were observed in control mice (PBS + ZIKV and MVA + ZIKV; Fig. 5A–D), whereas MVA-ZIKV vaccinated mice who had titers lower than control mice that was most likely due to the rapid control and suppression of ZIKV viral loads in the vaccinated mice.

Both PBS and MVA-control mice had detectable ZIKV-specific nAb-responses after ZIKV challenge and at the time point of death (8 dpc) with a mean titer of 1:170 and 1:217 respectively. Unlike the control mice, the MVA-ZIKV vaccinated mice survived ZIKV infection and till the end of the study and had a mean nAb-titer of 1:896 (Fig. 5E). Unlike the control mice that were euthanized at day 8 after ZIKV infection at which time they were sampled, the vaccinated mice did not suffer from either weight loss or display any clinical symptoms and were only euthanized at day 28. In order to avoid any impact on the outcome of ZIKV challenge, blood samples were not collected from the vaccinated mice at the day 8 time point.

To determine if ZIKV-specific nAb-responses contributed to protection immediately after ZIKV challenge, we performed passive serum transfer experiments. We first examined nAb-responses in IFNAR-/- mice after vaccination followed by ZIKV-infection 2-days later. On 6 dpc, low levels of ZIKV-specific nAb were detectable in MVA-control mice (mean PRNT_90_ titer of 1:115, Fig. 5F) that did not significantly differ from that of the MVA-ZIKV vaccinated mice (mean PRNT_90_ titer of 1:145 (Fig. 5F), suggesting that there was no anamnestic increase in nAb titers in the vaccinated mice in response to ZIKV-infection immediately after infection. However, we observed an increase in the mean nAb-titer by 10 dpc (mean PRNT_90_ titer of 1:266, Fig. 5F) in the vaccinated and protected mice suggesting an increased response to infection.

To confirm if ZIKV-specific nAb responses had a limited role in protection immediately after the challenge, serum was collected from each MVA-ZIKV vaccinated and MVA-only control group of mice at 6 dpc and pooled for passive transfer studies. Approximately 100 µl of pooled serum/mouse was injected IP into IFNAR-/- mice, followed by a lethal ZIKV challenge 24 h later. Mice in both the MVA-ZIKV vaccinated and MVA-control group showed signs of weight loss and neurological disease following challenge with ZIKV and succumbed to infection (Fig. 5G, Fig. S5C), suggesting a limited role for nAb responses in protection during the early stages of infection. In contrast, pooled sera from mice that had high nAb-titers at day 34 pv (mean PRNT_90_ titers of 1:1228 (Fig. 5H), fully protected mice with no apparent signs of weight loss or disease following infection with ZIKV (Fig. 5I, Fig. S5D).

Taken together, these results suggest that nAb responses induced immediately after vaccination were likely too low to afford any significant protection during the 1st week after rapid challenge when ZIKV replication and dissemination are high.

### ZIKV specific CD8+ T cell responses are detected early after MVA-ZIKV vaccination and correlate with rapid protection

**Fig. 6:**
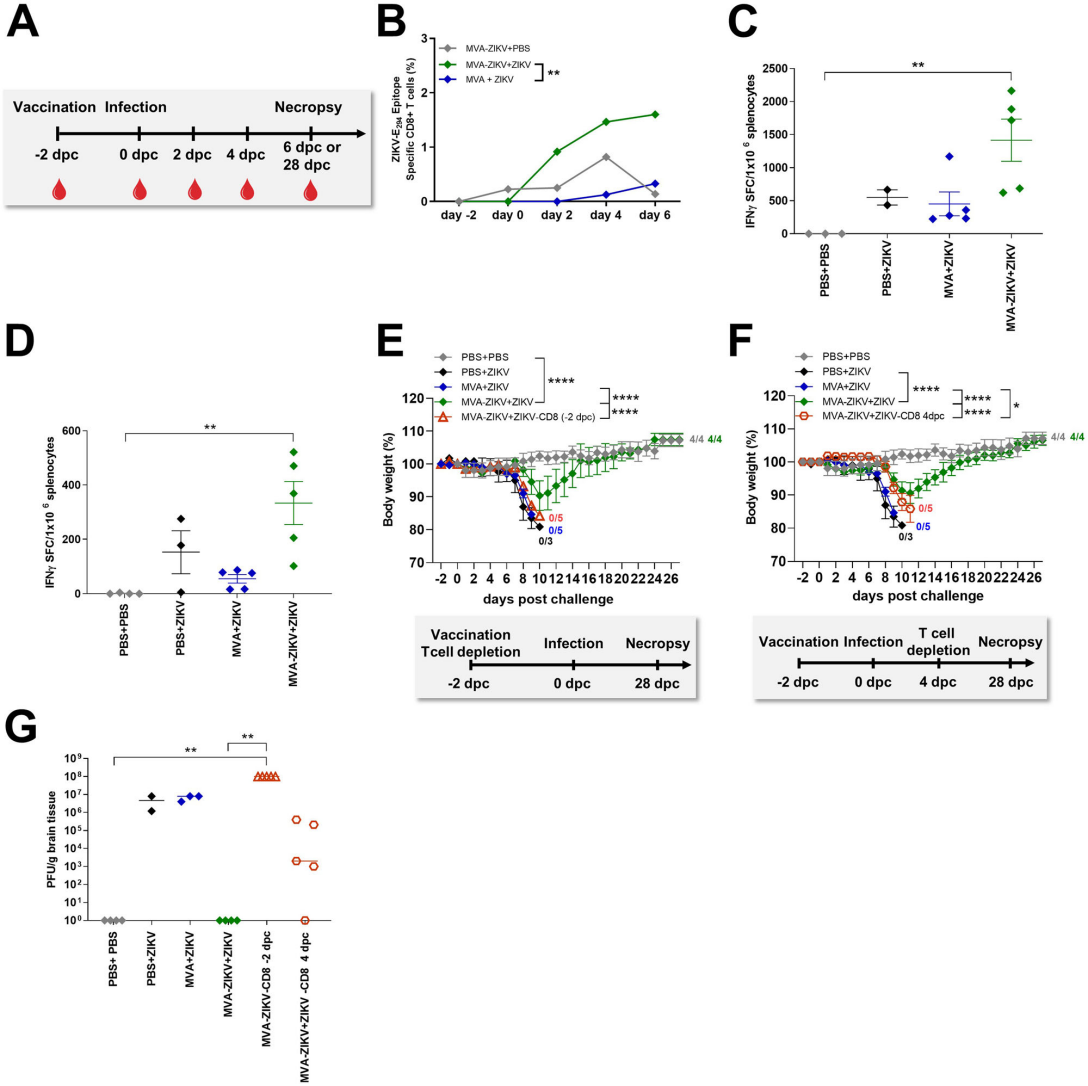
Rapid protection from MVA-ZIKV vaccination against lethal ZIKV challenge is lost in the absence of CD8+ T cells. **A** Study design: IFNAR-/- mice were vaccinated with MVA-ZIKV or empty MVA (10^8^ PFU) or PBS and challenged 2 days later with ZIKV or PBS. Blood (−2, 0, 2, 4, 6 dpc) and splenocytes (6 dpc) were prepared and analyzed for (**B**) the frequency of expanded E_294-302_-specific CD8+ T cells in IFNAR-/- mice or (**C**) IFN-γ SFC after stimulation with E_294-302_ peptide or (**D**) E_646-664_ peptide measured by ELISPOT assay 6 dpc. **E**–**G** IFNAR-/- mice depleted of CD8+ T cells at different time points after vaccination (*n* = 5, -2 dpc or 4 dpc) were challenged with ZIKV 2 days after intramuscular vaccination with 10^8^ PFU of MVA-ZIKV and compared to empty MVA (*n* = 5) or mock (PBS, *n* = 3) vaccinated animals used as controls. PBS-vaccinated + PBS-challenged mice (PBS + PBS, *n* = 4) were used as negative controls. Non-depleted MVA-ZIKV vaccinated mice (MVA-ZIKV + ZIKV, *n* = 4) were used as positive controls for emergency protection induced by the MVA-ZIKV vaccine. Body weight change was monitored daily for IFNAR-/- mice that had been depleted of CD8+ T cells (**E**) at -2 dpc (*n* = 5) or (**F**) 4 dpc (*n* = 5). **G** Brains were taken at the time of death and analyzed for the amounts of infectious ZIKV by PFU/gram tissue. Each dot indicates for a single mouse (**C**, **D**, **G**). The horizontal lines and associated error bars indicate for mean ± standard error of the mean (SEM). Differences between individual groups were analyzed by determining the area under the curve (AUC) prior to analysis by Kruskal-Wallis Test (**B**) or prior to analysis by one-way ANOVA (**E**, **F**) or single analysis by Kruskal-Wallis Test (**C**, **D**, **G**). Asterisks represent statistically significant differences between groups: * *p* < 0.05, ** *p* < 0.01, **** *p* < 0.0001.

Next, we examined ZIKV-E-specific T cell responses in the blood of MVA-ZIKV vaccinated mice and compared them to control groups (Fig.6A). ZIKV-E-specific CD8+ T cells were analyzed by flow cytometry using the IGVSNRDFV-Kb dextramer that specifically recognized the immunodominant E_294-302-_derived CD8+ T cell epitope^26^. As early as 2-days after vaccination but before infection (day 0 of ZIKV challenge), E_294-302-_epitope specific CD8+ T cells were readily detectable in MVA-ZIKV vaccinated mice (~0.022% of total CD8+ T cells; Fig. 6B) as compared to the MVA-only vaccinated control mice. After ZIKV-challenge, the frequencies of E_294-302_-specific T cells robustly expanded in MVA-ZIKV vaccinated mice (~0.9% at 2 dpc, ~1.46% at 4 dpc, and ~1.60% of total CD8+ T cells at 6 dpc; Fig. 6B) suggesting a substantial anamnestic response to vaccination. In contrast to MVA-ZIKV vaccinated mice, lower levels of E_294-302_-specific CD8+ T cells were detected in empty MVA-only control mice (~0.12% at 4 dpc, and ~0.32% at 6 dpc; Fig. 6B).

To confirm the role of T cell responses in protection from rapid challenge, we examined ZIKV-E-epitope specific CD8+ and CD4+ T cell responses in splenocytes at 6 dpc from MVA-ZIKV vaccinated mice using an IFN-γ ELISPOT-assay and compared them to control groups. Our results showed that ZIKV-infection was accompanied by a significant increase in the numbers of E_294-302_-epitope-specific CD8+ T cells in MVA-ZIKV vaccinated mice (~1420 SFC/million splenocytes; Fig. 6C) as compared to control mice (mean of 550 SFC/10^6^ splenocytes; Fig. 6C). Likewise, MVA-ZIKV vaccinated mice had high levels of E_646-662_-epitope-specific CD4+ T cell responses (mean 333 SFC/million splenocytes); Fig. 6D) as compared to control mice (mean of 152 SFC/million splenocytes; Fig. 6D).

ELISPOT responses aligned with flow cytometric analysis of T cells stained intracellularly for IFN-γ and TNF-α (Fig. S6A–F) following short-term stimulation with ZIKV-E-specific peptides. We also found higher frequencies of IFN-γ + CD8+ T cells in the MVA-ZIKV mice (~23.5%) as compared to the control groups (~4.6% for PBS + ZIKV, ~2.4% for MVA + ZIKV; Fig. S6A). The MVA-ZIKV vaccinated mice also had higher absolute numbers of IFN-γ + TNF-α + CD8+ T cells relative to the control groups (Fig. S6B). Moreover, MVA-ZIKV vaccinated mice had a higher frequency of cytokine-producing cells that expressed both IFN-γ and TNF-α (~38.6% Fig. S6C), suggesting that vaccination-induced poly-functional T cell responses. A substantial proportion of the activated CD4+ T cells in MVA-ZIKV vaccinated ZIKV-challenged mice also co-expressed TNF-α (~48% of cytokine-producing CD4+ T cells, Fig. S6F), suggesting that rapid protective vaccination with MVA-ZIKV induced potent ZIKV-E-specific CD4+ and CD8+ T cell responses that were significantly boosted immediately after infection.

To determine if ZIKV-specific T cell responses played a primary role in driving the rapid protective efficacy of that MVA-ZIKV vaccine, we depleted CD8+ T cells from IFNAR-/- mice at -2 dpc and 4 dpc using CD8+ T cell-specific depleting antibody. Successful depletion was confirmed by flow cytometry (Fig. S7A, B). As expected, non-depleted MVA-ZIKV vaccinated mice were fully protected from ZIKV infection, whereas all unvaccinated control mice experienced significant weight loss, including severe neurological disease and had to be euthanized at latest 10 dpc (Fig. 6E). Likewise, depletion of CD8+ T cells at day -2 prior to ZIKV challenge in MVA-ZIKV vaccinated mice was associated with severe disease and all mice succumbed to ZIKV infection, with a disease pattern and time to death similar to control animals (Fig. 6E, Fig. S8A). In contrast, depletion of CD8+ T cells at 4 dpc from MVA-ZIKV vaccinated mice resulted in delayed onset of disease; loss of body weight was observed 2-days later than the onset of body weight loss in unvaccinated controls (Fig. 6F, Fig. S8B). Nevertheless, CD8+ T cell depletion on 4 dpc was associated with 100% mortality within 12 dpc.

To determine if protection was associated with viral control, we analyzed ZIKV viral loads in the brains that were collected at the time of death. Brains of PBS and empty MVA-control mice had significantly high levels of ZIKV-infection (~1 × 10^7^ PFU; Fig. 6G) as compared to undetectable viral loads in MVA-ZIKV vaccinated mice (Fig. 6G). Depletions of CD8+ T cells at -2 dpc were associated with a dramatic increase in viral loads in the brains of MVA-ZIKV vaccinated mice (~1 x 10^8^ PFU). Likewise, depletion of CD8+ T cells at 4 dpc was accompanied by viral rebound (~8 × 10^5^ PFU), suggesting a loss of viral control in the absence of CD8+ T cells.

Taken together, our results suggest that ZIKV-specific CD8+ T cell responses were induced immediately after MVA-ZIKV vaccination and played a critical role in rapid protection against lethal ZIKV challenge.

### Rapid vaccination with MVA-ZIKV protects pregnant mice and fetus from lethal ZIKV challenge

**Fig. 7:**
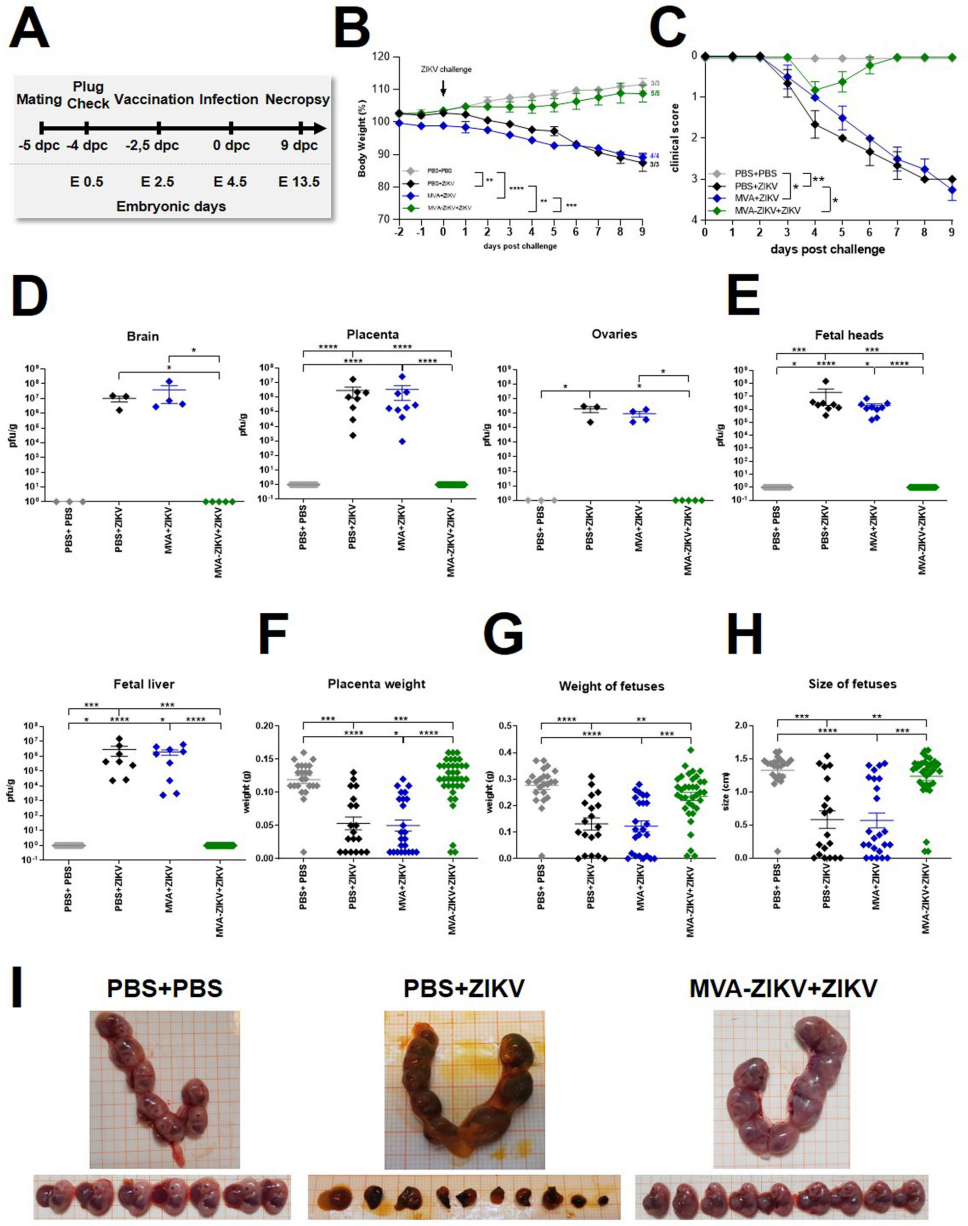
Rapid protection after MVA-ZIKV vaccination against lethal ZIKV challenge in pregnant mice. **A** Experimental scheme: 6–10 week-old female IFNAR-/- mice were mated. Successful mating was confirmed by plug check and pregnancy was determined by Ultrasound (US) examination. Positive mice were separated and immunized IM with 10^8^ PFU of recombinant MVA-ZIKV (*n* = 5) or empty MVA (*n* = 4) or PBS (*n* = 3) as control at −2 dpc (E 2.5). Mice were then challenged with 10^3^ PFU ZIKV SC at d0 (E 4.5) and monitored for symptoms and weight loss. PBS-vaccinated and PBS-challenged pregnant mice (PBS + PBS, *n* = 3) were used as controls. 9-days after challenge (E13.5) all dams were euthanized, and maternal and fetal organs were collected for analysis. Blood was taken at the end of the experiment. **B** Body weight change from -2 to 9 dpc. **C** Clinical symptoms are shown as score between 0 (no symptoms) and 4 (moribund/dead). Error bars represent standard error. **D** Maternal organs (brain, placenta and ovaries) were harvested at 9 dpc (E13.5) and analyzed for viral loads. Titers were determined by plaque assay and are shown as PFU/g of tissue. **E** Organs of fetuses (head and liver) were harvested at 9 dpc and analyzed for viral loads. **F** Placental weight and (**G**) weight and size of fetuses were determined at 9 dpc. **H** Representative images of uterus and fetuses collected at 9 pc (E13.5, endpoint) from mock-challenged (PBS + PBS), mock-vaccinated+ZIKV challenged (PBS + ZIKV), and MVA-ZIKV-vaccinated+ZIKV challenged groups (+ZIKV). Each dot indicates for a single mouse (**D**–**H**). The horizontal lines and associated error bars indicate for mean ± standard error of the mean (SEM). Differences between individual groups were analyzed by determining the area under the curve (AUC) prior to analysis by one-way ANOVA (**B**) or prior to analysis by Kruskal-Wallis Test (**C**) or single analysis by Kruskal-Wallis Test (**D**–**G**). Asterisks represent statistically significant differences between two groups: * *p* < 0.05, ** *p* < 0.01, *** *p* < 0.001 **** *p* < 0.0001.

**Fig. 8:**
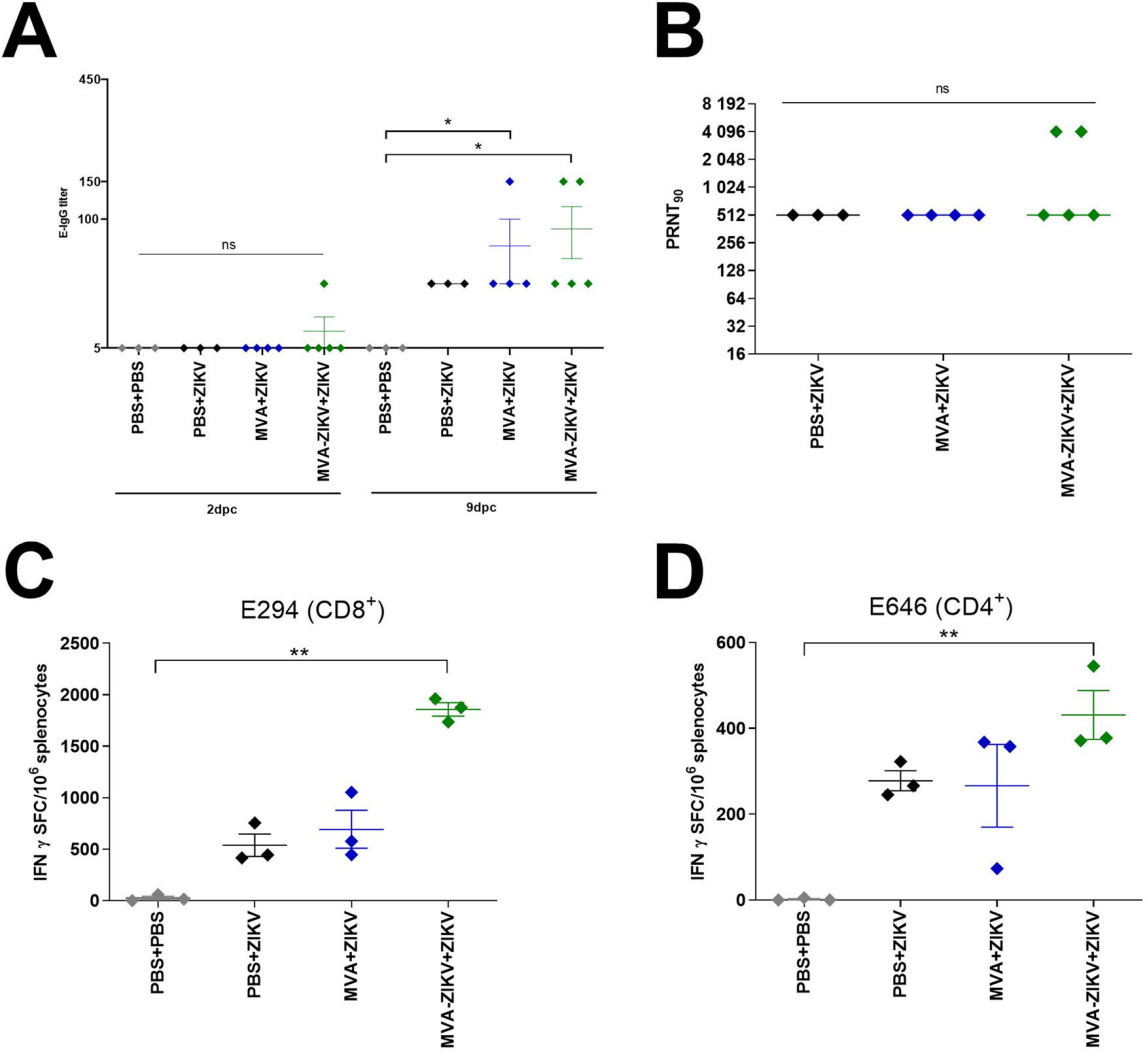
Immunogenicity of MVA-ZIKV vaccine in pregnant mice. **A**, **B** Activation of ZIKV-specific immune response in pregnant IFNAR-/- mice at 9 dpc (E13.5). **A** ZIKV-E-specific IgG-titers by ELISA. **B** ZIKV-neutralizing antibodies by plaque reduction assays (PRNT_90_). Dotted lines mark the upper and lower limit of detection. Activation of ZIKV-E specific T cells in pregnant IFNAR-/- mice at 9 dpc (E13.5). IFN-γ Spot Forming Counts (SFC) after stimulation with (**C**) E_294-302_ peptide measured by ELISPOT assay and (**D**) E_646-664_ peptide measured by ELISPOT assay. Each dot indicates for a single mouse. The horizontal lines and associated error bars indicate for mean ± standard error of the mean (SEM). Differences between the groups were analyzed by Kruskal-Wallis Test. Asterisks represent statistically significant differences between two groups: ns non-significant, * *p* < 0.05, ** *p* < 0.01.

To determine if rapid vaccination can be protective as an emergency vaccination, we tested the safety, immunogenicity, and efficacy of a single-shot MVA-ZIKV vaccination in pregnant mice 2 days before the lethal ZIKV challenge. Six–ten weeks old IFNAR-/- mice were mated and closely monitored for vaginal plug to confirm pregnancy and define embryonic day-0.5 (E0.5 = −4 dpc). At −2 dpc (E2.5), pregnant mice were vaccinated IM with MVA-ZIKV at a dose of 10^8^ PFU/mouse. Mock-vaccinated mice (PBS only and empty MVA vector only) served as controls. 2-days after vaccination (E4.5 = 0 dpc), all vaccinated and control mice were infected SC with 10^3^ PFU of ZIKV (Fig. 7A). All mice were closely monitored for morbidity and mortality (Fig. 7B, C). At 9 dpc (E13.5), all mice were sacrificed and analyzed for viral loads, fetal development and immune responses (Fig. 7, Fig. 8). Control pregnant mice displayed signs of ZIKV-disease starting 5 dpc (E9.5) which progressively increased in severity with severe neurologic disease with characteristic clinical symptoms, including partial or complete paralysis of one or both hind limbs, reduced activity, and scruffy fur reaching out a cumulative score of 3.5 until the end of the experiment (Fig. 7B, C). We did not detect the development of ZIKV-specific disease in MVA-ZIKV vaccinated dams, though a mild clinical score of 1 characterized by ruffled fur was detected at 1 dpc that resolved quickly (Fig. 7C). In contrast to the MVA-ZIKV vaccinated pregnant mice that were fully protected from clinical disease without an apparent loss of body weight (Fig. 7B, C), control pregnant mice experienced a substantial (10%) loss of body weight starting at 6.5 dpc (E10.5, Fig.7B).

To examine if vaccination with MVA-ZIKV was associated with clearance of the ZIKV infection, we examined the levels of viral loads in the dams (brain, placenta, ovaries) and fetuses. At 9 dpc (E13.5), we detected high ZIKV viral loads in the brain, placenta, ovaries of control pregnant mice (Fig.7D), but little or no detectable ZIKV in the tissues of MVA-ZIKV vaccinated mice. Like the pregnant dams, the fetuses from control mice were found to have substantial levels of infectious ZIKV, whereas fetuses from MVA-ZIKV vaccinated dams (Fig. 7E) had no detectable ZIKV.

Next, we examined the effect of ZIKV infection on fetal development and morphological defects in MVA-ZIKV vaccinated mice and compared them to control mice. In control dams, the weights of the placentas were significantly reduced as compared to MVA-ZIKV vaccinated dams indicating severe effects of maternal ZIKV infection (Fig. 7F). Likewise, the sizes and weights of fetuses from control dams were significantly reduced in comparison to fetuses from MVA-ZIKV vaccinated mice (Fig. 7G). Of note, in control dams most fetuses were resorbed while there were no resorbed fetuses observed in MVA-ZIKV vaccinated dams (Fig. 7I).

Control of infection was associated with the induction of both nAb and T cell responses. 2 dpc, titers of ZIKV-E binding antibodies were only detected in one MVA-ZIKV vaccinated mouse while all other mice did not mount ZIKV-E binding antibodies. 9 dpc, ZIKV-E-binding antibodies significantly increased in MVA-ZIKV vaccinated mice (mean titer of 1:90). MVA-control vaccinated mice had significantly reduced titers with a mean titer of 1:50. (Fig. 8A). Control pregnant mice had ZIKV specific nAb titers of 1:512 at 9 dpc as compared to a titer of 1: 1945 in the MVA-ZIKV vaccinated (Fig. 8B). Next, we characterized ZIKV-E-specific CD8+ (E_294-302_-epitope) and CD4+ (E_646-662_-epitope) T cells at 9 dpc in splenocytes using an IFN-γ ELISPOT assay. Infection with ZIKV was accompanied by a substantial induction of E_294-302_-epitope-specific CD8+ T cells in PBS only (~538 SFC/million splenocytes) and MVA only (~692 SFC/million splenocytes) control dams (Fig. 8C). Vaccination with the MVA-ZIKV vaccine was associated with a significant increase in E_294-302_-epitope specific CD8+ T cells (~1922 SFC/million splenocytes; Fig. 8C) as compared to the control dams. We observed a similar trend in ZIKV specific CD4+ T cell responses with MVA-ZIKV vaccinated mice displaying significantly higher levels of E_646-662_-epitope specific CD4+ T cells (~431 SFC/10^6^ splenocytes; Fig. 8D) as compared to control mice (~277 SFC/million splenocytes) suggesting the vaccinated dams mounted a significant anamnestic response to ZIKV infection.

## Discussion

Zika virus (ZIKV) infection during pregnancy poses a significant threat to the fetus thereby endangering vast numbers of pregnant women who live in regions where ZIKV is endemic. Strategies that can protect the fetus from ZIKV infection are essential to continue a healthy pregnancy without adverse effects on the newborn. Here, we demonstrate that vaccination with a recombinant MVA candidate-vaccine expressing ZIKV-prME-protein (MVA-ZIKV) mediates rapid protection against neurological ZIKV infection as well as in utero-transmission in the IFNAR-/- mouse model. Importantly, we show that ZIKV-specific CD8+ T cells play a key role in protection against ZIKV infection following rapid challenge.

In vivo, no ZIKV was detectable in the organs of either non-pregnant- and pregnant-vaccinated IFNAR-/- dams or fetuses suggesting that a rapid emergency vaccination during early pregnancy could potentially prevent lethal infection and rapidly clear the virus. Shan et al. demonstrated that vaccination with a live-attenuated ZIKV vaccine 10-days prior to the ZIKV challenge induced robust protection after a single immunization^27^. Our studies suggest that this protection was evident as early as 2 days after infection.

Interestingly, rapid protection was associated with significantly higher frequencies of ZIKV-E_294-302_-specific CD8+ T cells as early as 2 dpc, suggesting that CD8+ T cell responses played a key role in mediating the rapid protection we observed. Earlier studies have shown that E_294-302_ was an immunodominant determinant of the ZIKV-specific CD8+ T cell response in C57BL/6 mice, although other T cell specificities likely contribute to the protective immunity, we observed^28,29^. These findings were confirmed in CD8+ T cell-depleted MVA-ZIKV vaccinated IFNAR-/- mice that were found to have significantly higher viral loads (Fig. 6G), as compared to undetectable viral loads in MVA-ZIKV vaccinated mice that were not depleted of CD8+ T cells. Likewise, later depletion of CD8+ T cells at 4 dpc in MVA-ZIKV vaccinated IFNAR-/- mice was associated with loss of viral control and death as compared to total control of tissue viral loads and survival in un-depleted MVA-ZIKV vaccinated IFNAR-/- mice.

Numerous studies have documented a clear protective role for CD8+ T cells in controlling ZIKV and other flavivirus infections^26,30–34^. A recent study using a pre-clinical Aripo/ZIKV vaccination in T cell deficient mice that lack T cell responses demonstrated complete loss of protection after ZIKV challenge supporting a key role for CD8+ T cells protection against ZIKV^35^. The essential role of T cells for ZIKV protection has been further demonstrated using a DNA-Vaccine platform in different ZIKV mouse models^36^. As with the non-pregnant MVA-ZIKV vaccinated IFNAR-/- mice, we observed a significant increase in ZIKV-E-_294-302_-epitope-specific CD8+ T cell responses in MVA-ZIKV vaccinated IFNAR-/- pregnant dams at 9 dpc (Fig. 8C-D). We were unable to sample these mice at early time points due to the potential for adverse effects on their pregnancy. Our results suggest that CD8+ T cell responses induced by vaccination and anamnestically boosted immediately after infection is strongly protective during pregnancy. Though we only evaluated immune responses against an immunodominant epitope, it is likely that vaccination induced a polyclonal response that played a role in protection. How well these epitope specific responses readily translate to humans is yet to be determined as epitope specific responses are likely to vary between mice and humans given the differences in MHC class I restriction. However, as a proof-of-concept our studies strongly support a role of CD8+ T cell responses in protection, and induction of a polyclonal CD8+ T cell response against immunodominant epitopes in humans could protect during pregnancy.

We cannot rule out a role for nAb responses in protection as 2/5 mice (Fig. 8B) had higher nAb-titers even though there was no significant difference in nAb-titers between MVA-ZIKV vaccinated IFNAR-/- pregnant dams and control mice at 9 dpc. Whether nAb responses played a role in early protection in MVA-ZIKV vaccinated mice is difficult to determine as nAb-responses were not detected at 6 dpc. These results align with those reported by Shan et al.^37^, who had immunized mice 10 days before the challenge and reported induction of strong nAb-responses that correlated with protection. Unlike Shan et al.^27^, we immunized mice 2 days prior to challenge. Similar to non-pregnant IFNAR-/- mice, we noted a delayed emergence of nAb-responses in the pregnant IFNAR-/- mice suggesting that nAb-responses may contribute to protection albeit at later stages of infection, likely by limiting the spread of ZIKV to maternal tissues and fetuses. Passive transfer studies were found to support this hypothesis; serum obtained from MVA-ZIKV vaccinated mice at 6 dpc transferred to IFNAR-/- mice prior to challenge failed to protect mice from infection (Fig. 5G). On the other hand, serum obtained at 6 dpc using a late ZIKV-challenge 28 days after initial vaccination, completely protected IFNAR-/- mice from ZIKV-challenge, suggesting that nAb-responses likely play a critical role in protection at the later stages of infection. These findings are in line with previous studies^2,10,14,37^.

The delay in the emergence of nAb-responses is not surprising given the short duration of 2 days between vaccination and challenge and the time needed for the activation and differentiation of B cells following vaccination. Zika viral loads peak between 2 and 4 dpc^38^. As such, delayed nAb-responses are likely too little and too late to effectively prevent infection and mediate viral clearance within a few days after vaccination and challenge that is in line with earlier studies demonstrating an essential role of nAb-responses during later stages after infection^6,9,39,40^. Induction of cross-reactive antibody responses, however, needs to be interpreted with caution. Numerous studies in both non-human primates^41–43^ and humans^44–47^ have demonstrated that antibody responses induced by prior ZIKV infection leads to an enhanced risk for severe Dengue (DENV) infection. Vaccines that can induce potent CD8 T cell responses can overcome some of these concerns. Elong et al. used an NS3 based vaccine to demonstrate that CD8+ T cells induced by the vaccine protected mice from lethal challenge^48,49^ showed that a DNA vaccine that expressed ubiquitinated ZIKV NS3 primarily induced CD8+ T cells that protected in pregnant IFNAR1-/- mice and their fetuses from ZIKV infection. Likewise, Burg et al.^50^ used a recombinant Listeria monocytogenes vaccine encoding a single MHC Class I restricted ZIKV epitope and demonstrated that vaccination significantly reduced viral burden in mice. Numerous other studies have reported that vaccines that can induce potent ZIKV specific CD8+ T cell responses can afford significant protection from lethal ZIKV infection^51,52^ recently demonstrated that CD8+ T cells were capable of effectively controlling flavivirus infections in humans even in the absence of nAb responses. Taken together, results from our studies suggest that rapid vaccination with the MVA-ZIKV vaccine was efficacious in both immunodeficient non-pregnant and pregnant mice, with a predominant role of ZIKV specific CD8+ T cell responses in mediating the rapid clearance of ZIKV and protecting the fetus from an adverse outcome. Whether MVA based vaccination will be safe in pregnant women is yet to be determined though our results suggest that MVA-ZIKV vaccination had no untoward effect on either the dams or fetuses. Larger studies in pregnant NHP models are needed to better assess the translational efficacy of rapid MVA-ZIKV vaccination to prevent ZIKV infection and protect the fetus in pregnant macaques.

## Materials and methods

### Experimental design

The overall goal of this effort was to determine the rapid immunogenicity and efficacy conferred by an MVA-ZIKV vaccine candidate in a lethal mouse model for ZIKV. The MVA-ZIKV candidate vaccine was evaluated in non-pregnant and pregnant mice. MVA-ZIKV-induced immune responses were analyzed in detail to identify immunological mechanisms of rapid protection.

### Cells and viruses

Primary chicken embryonic fibroblasts (CEF) were prepared from 10 to 11-day-old chicken embryos (SPF eggs, VALO, Cuxhaven, Germany) as previously described^53^. Vero cells (African green monkey kidney, ATCC CCL-81) were maintained in Dulbecco’s Modified Eagle’s Medium (DMEM). Human HeLa cells (ATCC CCL-2) were maintained in Minimum Essential Medium Eagle (MEM) (Sigma-Aldrich, Taufkirchen, Germany), 7% FBS, and 1% MEM non-essential amino acid solution. Plaque-purified Zika virus (ZIKV) isolate H/PF/2013 (EVAg, clinical isolate, French Polynesia 2013, GenBank Sequence Accession: KJ776791) was propagated on Vero cells. Viral titers were determined by plaque assay and titrated, with values reported in PFU^54^.

### MVA-ZIKV vaccine

MVA virus was used as a backbone virus to construct recombinant MVA expressing the ZIKV- prM and E target gene sequences (MVA-ZIKV) as described previously^53,55^. The gene fragments encoding prM and E protein from Amino acid 126-790 without M-signalpeptide from ZIKV Yap 2006 strain (GenBank accession number AEN75266.1) were obtained as a synthetic, poxvirus-codon optimized gene sequence (Genewiz, Leipzig, Germany) and cloned into the MVA transfer plasmid pIIIH5red-K1L under transcriptional control of the synthetic vaccinia virus early/late promoter PmH5 to obtain the MVA expression plasmid pIIIH5red-K1L- ZIKV-prME. Cells were infected at an MOI of 0.05 with MVA-ZIKV or empty MVA (MVA) and viral titers were determined using plaque assay^55^.

### In vitro characterization of recombinant MVA-ZIKV

Genetic identity and genetic stability of vector viruses was confirmed by polymerase chain reaction (PCR) using viral DNA. To confirm the genetic integrity and proper insertion of the ZIKV-prME gene within the MVA genome we performed PCR analysis of the viral genomic DNA using specific oligonucleotide primers targeting sequences adjacent to the MVA deletion site I-VI^55^. As a control for equal amounts of viral DNA, a second PCR amplified C7L gene sequences in the MVA genome, was used^55^. Two different PCRs were used to assess the integrity of the ZIKV-prME gene sequence inserted in the MVA-ZIKV genome. For this, two pairs of primers specifically amplifying the ZIKV-prME gene sequences were used (Primer pair 1: forward GCCATTCTCTTGGCACCTCT, reverse TTCCATTACCTTGGCACGCT, primer pair 2: forward ATGTCACCAGGCTCCCTTTG, reverse GTGTACGGAACCTGCCATCA, Eurofins Genomics, Ebersberg, Germany). The replicative capacity of recombinant MVA-ZIKV was tested in multi-step-growth experiments on monolayers of CEF and HeLa cells grown in 6-well-tissue-culture plates. Viruses were inoculated at MOI 0.05, harvested at 0, 4, 8, 24, 48, and 72 h after infection, and titrated on CEF monolayers to determine infectivities in cell lysates in PFU.

### Western blot analysis of recombinant protein

To monitor the production of the recombinant ZIKV-E-protein expressed by MVA, CEF cells were infected at MOI 0.5. Proteins from lysates were separated by electrophoresis in a sodium dodecyl sulfate (SDS)-10% polyacrylamide gel (SDS-PAGE) and transferred to a nitrocellulose membrane by electroblotting. The membrane was blocked in a phosphate-buffered saline (PBS) buffer containing 5% milk (AppliChem GmbH, Darmstadt, Germany) and 0.1% Tween-20 (Sigma-Aldrich, Taufkirchen, Germany) and was incubated overnight with a primary, monoclonal antibody targeting the Flavivirus-E protein (1:5000; Absolute Antibody, Wilton, UK). The membrane was washed with PBS/0.1% Tween-20 and incubated with an anti-mouse IgG antibody (1:5000; Agilent Dako, Glostrup, Denmark) conjugated to HRP. The blot was washed and developed using SuperSignal® West Dura Extended Duration substrate (Thermo Fisher Scientific, Planegg, Germany). Chemiluminescence was visualized using the ChemiDoc MP Imaging System (Bio-Rad, Munich, Germany).

### Immunostaining for recombinant protein

Vero cells were infected with MVA-ZIKV at a MOI of 0.5 or non-recombinant MVA and incubated for 16 h at 37 °C. Cells were fixed with 4% paraformaldehyde (PFA) for 10 min on ice, and washed with PBS. Cells were, permeabilized with 0.1% Triton X-100 (Sigma-Aldrich) and probed with a monoclonal antibody against the E protein (1:1000; ZIKV-E, Biozol, Germany). Nuclei were stained with 1 µg/ml of 4,6-diamidino-2-phenylindole (DAPI) (Sigma-Aldrich) and cells were analyzed using the Keyence BZ-X700 microscope (Keyence, Neu-Isenburg, Germany) with a ×100 objective.

### Animals

C57BL/6 mice and IFNAR-/- mice backcrossed more than 20-fold on the C57BL/6 background were bred under specified-pathogen-free (SPF) conditions at the animal facility of LMU Munich and housed in isolated cage units (Tecniplast, Hohenpeißenberg, Germany). 6–8 weeks old, healthy animals in good condition were used for the animal experiments. Animals were assigned to study groups randomly while ensuring equal distribution of sex and bodyweight. For pregnancy experiments, only female animals were used. All experiments were performed in compliance with the European and national regulations for animal experimentation (European Directive 2010/63/EU; Animal Welfare Acts in Germany) and approved by the government of Upper Bavaria, corresponding to the standards of IACUC.

### Immunizations and infection experiments in mice

Male and female Mice were vaccinated with 10^8^ PFU of MVA-ZIKV, empty MVA, or PBS into the quadriceps muscle of the left hind leg without anesthesia. Mice were infected with 10^3^ PFU of ZIKV-strain H/PF/2013 (EVAg, clinical isolate, French Polynesia 2013, GenBank Sequence Accession: KJ776791) diluted in 50 µl of physiological saline subcutaneously via footpad infection in the left hind leg. Control mice were mock infected with PBS. Signs of illness, weight loss, clinical disease, and survival were monitored daily as established before^56^ until the end of the experiment day (28 dpc). 500 µg/kg body weight (BW) Medetomidin +5 mg/kg BW Midazolam was used to anaesthesize mice for subcutaneous ZIKV infections for a gentle and precise injection of the viral suspension. To euthanize the animals at the end of the experiment, the animals were anesthetized with isoflurane using a vaporizer in a whole-body chamber and then exsanguinated via cardiac puncture.

### Immunization and infection in pregnant mice

Female 6–10-week-old IFNAR-/- mice were paired 1:1 with males for 24 h for mating. Mice were then checked for a genital plug to confirm successful mating and positive females were separated. Mice were examined via ultrasound 4-days later to confirm pregnancy^54^. Pregnant mice were vaccinated with 10^8^ PFU of the MVA-ZIKV, MVA only or PBS as described above via intramuscular injection into the left hind leg without anesthesia. 2-days later mice were infected with 10^3^ PFU of ZIKV as described before. Mice were checked daily for survival, clinical condition, and weight loss. 9 dpc mice were euthanized and fetuses and maternal organs (placenta, ovaries, brain) were harvested to determine size, weight, and ZIKV.

500 µg/kg body weight (BW) Medetomidin +5 mg/kg BW Midazolam was used to anaesthesize mice for subcutaneous ZIKV infections for a gentle and precise injection of the viral suspension. To euthanize the animals at the end of the experiment, the animals were anesthetized with isoflurane using a vaporizer in a whole-body chamber and then exsanguinated via cardiac puncture.

### Clinical disease

Signs of illness, weight loss, clinical disease and survival were observed daily. To characterize clinical disease, mice were monitored daily and were allocated to one of the following categories of ZIKV specific disease as established by Lazear et al.^44^: no disease, hind limb weakness or disrupted righting reflex, partial hind limb paralysis or toe knuckling, complete paralysis of one hind limb, complete paralysis of both hind limbs, complete paralysis of all four limbs, or moribund or dead; mice with a disease score of complete paralysis of two or more hind limbs or moribund as measured by weight loss of more than 20% of initial body weight loss were euthanized.

### Antigen-specific IgG/IgM ELISA

In mice, ZIKV-specific serum IgM or IgG titers were analyzed using the rec ZIKV-E-protein or live ZIKV. Diluted serum samples (1:10 in sample buffer) were first incubated at 37 °C for 30 min in microplate wells coated with ZIKV-E-protein or live ZIKV. For detection, an enzyme-labeled anti-mouse IgG was then added to the wells in a second incubation step (37 °C, 30 min). TMB substrate was added to the wells and the reaction was stopped after 15 min and the OD was determined at 450 nm light, and semi-quantitative evaluations were performed by using the ratio values.

### Plaque Reduction Neutralization Test (PRNT_90,_ PRNT_50_)

Serum samples were tested for their capacity to neutralize ZIKV strain H/PF/13 using plaque reduction neutralization test (PRNT). PRNT_90_ or PRNT_50_ titers were determined using sera from mice. Sera were heat-inactivated for 30 min at 56 °C, serially diluted two-fold in 96-well plates, and 200 PFU of ZIKV strain H/PF/13 were added to the same volume of each serum dilution. After incubating for 2 h at 37 °C, the mixture was used to inoculate Vero cells (70% confluent) in 24 well plates. After 1 h, 2% carboxymethyl cellulose (CMC) mixed in a 1:1 ratio with 2× cell culture medium was added to each well as overlay, and cells were incubated at 37 °C. After 72 h, overlay medium was removed, and crystal violet was used to stain cells to identify plaques. PRNT_90_ was defined as the reciprocal of the serum dilution that inhibited ≥90% of tested virus plaque formation. PRNT_50_ was defined as the reciprocal of the serum dilution that inhibited ≥50% of tested virus plaque formation.

### T-cell analysis by Enzyme-Linked Immunospot (ELISPOT)

Splenocytes were harvested, washed, and resuspended in RPMI-10. IFN-γ ELISPOT assay (Mabtech ELISpot, Biozol, Eching, Germany) was performed as per manufacturer’s instructions. Briefly, 2 × 10^5^ splenocytes/100 µl were stimulated with individual ZIKV-specific peptides (E_294-302_ (IGVSNRDFV)^26^, E_646-662_ (GRLITANPVITESTE)^57^), at a concentration of 2 µg/mL in RPMI-10. Unstimulated cells and cells stimulated with phorbol myristate acetate (PMA; 10 ng/ml)/ionomycin (500 ng/ml, Sigma-Aldrich) or vaccinia virus peptide B8R_20–27_ (TSYKFESV) were used as positive controls. After incubation for 48 h, plates were stained, and spots were counted and analyzed using an automated and analyzed using ELISPOT plate reader (A.EL.VIS Eli. Scan, A.EL.VIS ELISPOT Analysis Software, Hannover, Germany).

### T-cell analysis in blood using FACS analysis

Mice were bled on -2, 0, 2, 4, and 6 dpc and 50 µl of heparinized blood was preincubated with 10 µl of MVA- or ZIKV-CD8+ specific dextramers (Immudex, Virum, Denmark) for 15 min at room temperature. After preincubation, anti-mouse CD3 phycoerythrin (PE)-Cy7 (clone 17A2, 1:100, Biolegend, London, United Kingdom), anti-mouse CD4 phycoerythrin (PE)-Cy7 (clone GK1.5, 1:600, Biolegend) and anti-mouse CD8α Alexa Fluor 488 (clone 53-6.8, 1:300, Biolegend,) using 50 µl/sample diluted in FACS buffer was added. After incubation, samples were lysed with Red Blood Cell Lysis Buffer (Sigma-Aldrich) and resuspended in FACS Buffer. Data was acquired by the MACSQuant VYB Flow Analyzer (Miltenyi Biotec, Bergisch Gladbach, Germany) and analyzed using FlowJo (FlowJo LLC, BD Life Sciences, Ashland, OR, USA).

### T cell depletion

Mice were depleted of CD8+ T cells by IP administration of mouse monoclonal antibodies obtained from Harlan Bioproducts, Indianapolis, USA^58,59^. To deplete CD8+ T cells before immunization for day -2 pc, mice were treated with ~100 mg of anti-CD8 (clone 2.43) antibody on days -4, and -3. To deplete CD8+ T cells after immunization for day 4 pc, the mice were treated with the depleting antibody on day 2, and day 3 after challenge. CD8+ T cell depletion was confirmed using flow cytometry.

### T cell analysis to confirm CD8+ T cell depletion in blood using FACS analysis

Mice were bled on day -2 or and 4 pc, and 50 µl of heparinized blood was incubated with anti-mouse CD3 phycoerythrin (PE)-Cy7 (clone 17A2, 1:100, Biolegend, Biolegend, London, United Kingdom), anti-mouse CD4 phycoerythrin (PE)-Cy7 (clone GK1.5, 1:600, Biolegend, Biolegend, London, United Kingdom) and anti-mouse CD8α Alexa Fluor 488 (clone 53–6.8, 1:300, Biolegend, Biolegend, London, United Kingdom) using 50 µl/sample diluted in FACS buffer was added. After 30 min on ice, blood samples were incubated with Red Blood Cell Lysis Buffer (Sigma-Aldrich, Taufkirchen, Germany). Cells were washed and resuspended in FACS Buffer. Data was acquired by the MACSQuant VYB Flow Analyzer (Miltenyi Biotec, Bergisch Gladbach, Germany) and analyzed using FlowJo (FlowJo LLC, BD Life Sciences, Ashland, OR, USA).

### T cell analysis by Intracellular Cytokine Staining (ICS)

The detailed methods for intracellular cytokine staining (ICS) were described previously^41^. Briefly, whole mouse splenocytes were diluted in RPMI-10 and plated onto 96-well-U-bottom plates using 10^6^ cells/well. Cells were stimulated with 8 µg/ml E_294-302_ (IGVSNRDFV) or E_646-662_ (GRLITANPVITESTE) peptide or Vaccinia virus peptide B8R_20–27_ (TSYKFESV) to analyze ZIKV-E CD4+ and CD8+ T cells or MVA specific CD8+ T cells. Splenocytes stimulated with PMA (10 ng/ml) plus ionomycin (500 ng/ml) served as positive controls and RPMI alone was used as a negative control. After 2 h at 37 °C, Brefeldin A (Biolegend, San Diego, CA, USA) was added according to the manufacturer’s instructions and stimulated cells were further maintained for 4 h at 37 °C. After stimulation, cells were washed with FACS buffer (MACSQuant Running Buffer, Miltenyi Biotec, Bergisch Gladbach, Germany, plus 2% FBS) and stained extracellularly with anti-mouse CD3 phycoerythrin (PE)-Cy7 (clone 17A2, 1:100, Biolegend), anti-mouse CD4 Brilliant Violet 421 (clone GK1.5, 1:600, Biolegend, San Diego, CA, USA), anti-mouse CD8α Alexa Fluor 488 (clone 53-6.8, 1:300, Biolegend), and purified CD16/CD32 (Fc block; clone 93, 1:500, Biolegend, San Diego, CA, USA) using 50 µl/well diluted in FACS buffer for 30 min on ice. After staining and washing, cells were incubated with 100 µl/well of the fixable dead cell viability dye Zombie Aqua (1:800, Biolegend, San Diego, CA, USA) diluted in PBS for 30 min on ice. Cells were then washed, fixed with 100 µl/well of Fixation Buffer (Biolegend, San Diego, CA, USA) for 20 min at room temperature, washed again, resuspended in 200 µl/well of FACS buffer and stored overnight at 4 °C. Next, cells were permeabilized using Intracellular Staining Permeabilization Wash Buffer (Perm Wash buffer; Biolegend; dilution 1:10) and stained intracellularly in 100 µl/well of anti-mouse IFN-γ (clone XMG1.2, 1:200, Biolegend, San Diego, CA, USA) plus anti-mouse TNF-α (clone MP6-XT22, 1:200, Biolegend, San Diego, CA, USA) diluted in Perm Wash buffer for 30 min at room temperature. Thereafter, cells were washed with Perm Wash buffer and resuspended in FACS buffer. Prior to analysis, samples were filtered through a 50-μM-nylon mesh (Sefar Pty Ltd., Huntingwood, NSW, Australia) into 5 ml round bottom FACS tubes (Sarstedt, Nümbrecht, Germany). For each antibody, single color controls were prepared using OneComp eBeads™ Compensation Beads (eBioscience, Thermo Fisher Scientific, Waltham, Massachusetts, USA) and cells for the viability dye Zombie Aqua. Data was acquired by the MACSQuant VYB Flow Analyzer (Miltenyi Biotec, Bergisch Gladbach, Germany) and analyzed using FlowJo (FlowJo LLC, BD Life Sciences, Ashland, OR, USA).

### Determination of ZIKV loads in mouse organs

Organs were removed under aseptic conditions and immediately frozen and subsequently thawed, weighed, and homogenized using 0.1 g of organ material with 1 ml PBS in a microtube (Retsch Tissue Lyser, Qiagen GmbH, Hilden, Germany). Tubes were centrifuged for 1 min at 1500 rpm and 4 °C. Supernatants were taken and stored at −80 °C. Viral titers in organ supernatants were determined by plaque assays as described previously^54^ and reported as PFU/g of tissue.

### Statistical analysis

Data were prepared using GraphPad Prism 9.0.0 (GraphPad Software Inc., San Diego CA, USA) and expressed as mean ± standard error of the mean (SEM). Data were analyzed by two tailed Mann-Whitney test and Wilcoxon test to compare two groups and one-way ANOVA and Kruskal-Wallis test to compare three or more groups. Additional information can be found in the respective figure legend. A *P* < 0.05 was used as the threshold for statistical significance.

## Supplementary information

Supplementary information

## Data Availability

All data needed to evaluate the conclusions in this paper are present in the paper and/or the Supplementary Materials. The datasets and any unique materials used and/or analyzed during the current study available from the corresponding author on a reasonable request. The cDNA sequences coding for the prM and E protein from Aminoacid 127–790 of the ZIKV Yap 2007 isolate is available on GenBank using the accession number: EU545988.1YYYY.
