# Supplementary material for: Efficacy of emergency maternal MVA-ZIKV vaccination in a rapid challenge model of lethal Zika infection
Source: NPJ Vaccines. 2025 Mar 5;10:44. doi: 10.1038/s41541-025-01094-0 (PMC11882785; doi:10.1038/s41541-025-01094-0)
Supplement: Supplementary file 1 — Supplementary information [file 41541_2025_1094_MOESM1_ESM.pdf]

Supplementary Materials for

Efficacy of emergency maternal MVA-ZIKV vaccination in a rapid challenge model of lethal Zika infection

Asisa Volz *et al.*

*Corresponding author. Email: Asisa.Volz@tiho-hannover.de

This PDF file includes:

Supplementary Figures:

1. ZIKV-prME gene encoding sequence used for insertion in MVA-deletion site III.
2. Molecular analysis of the MVA-ZIKV-genome.
3. ZIKV-specific memory T cells induced after MVA-ZIKV vaccination in IFNAR-/- mice without ZIKV challenge infection.
4. Neutralizing antibodies after vaccination with MVA-ZIKV and lethal ZIKV challenge 28 day later.
5. Clinical disease after ZIKV challenge infection in IFNAR-/-mice.
6. Activation of ZIKV-specific T cells. Groups of IFNAR-/- mice (n = 2 - 4) were vaccinated with 10^8 PFU of MVA-ZIKV and challenged with ZIKV 2 days later.
7. Depletion of CD8+ T cell as confirmed by flow cytometry.
8. Clinical disease after ZIKV challenge infection in MVA-ZIKV vaccinated and CD8+ T cell-depleted IFNAR-/-mice.
9. Gating strategy used for ICS
10. – 12. Original and unprocessed scans of blots and microscopy images

Supplementary Figure 1:

acc**ATG**ACTAGACGTGGGAGTGCATACTATATGTACTTGGACAGAAACGATGCTGGaGAGGCCAT
ATCTTTcCCAACCACATTGGGaATGAATAAGTGTTATATACAGATCATGGATCTTGGACACATGTG
TGATGCCACCATGAGCTATGAATGCCCTATGCTGGATGAGGGaGTGGAACCAGATGACGTCGAT
TGTTGGTGCAACACGACGTCAACTTGGGTTGTGTACGGAACCTGCCATCACAAgAAgGGTGAA
GCACGGAGATCTAGAAGAGCTGTGACGCTCCCCTCCCATTCCACCAGGAAGCTGCAAACGCGG
TCGCAAACCTGGTTGGAATCAAGAGAATACACAAAGCACTTGATTAGAGTCGAgAATTGGATAT
TCAGGAACCCTGGCTTCGCGTTAGCAGCAGCTGCCATCGCTTGGCTTTTGGGAAGCTCAACGA
GCCAgAAAGTCATATACTTGGTCATGATACTGCTGATTGCaCCaGCATACAGCATCAGGTGCATAG
GAGTCAGCAATAGGGACTTTGTGGAAGGTATGTCAGGTGGGACTTGGGTTGATGTTGTCTTGG
AACATGGAGGTTGTGTCACCGTAATGGCACAGGACAAACCGACTGTCGACATAGAGCTGGTTA
CAACAACAGTCAGCAACATGGCGGAGGTAAGATCCTACTGCTATGAGGCATCAATATCAGACAT
GGCTTCTGACAGCCGCTGCCCAACACAAGGTGAAGCCTACCTTGACAAGCAATCAGACACTCA
ATATGTCTGCAAAAGAACGTTAGTGGACAGAGGCTGGGGAAATGGATGTGGACTaTTTGGCAA
AGGGAGCCTGGTGACATGCGCTAAGTTTGCATGCTCCAAGAAgATGACCGGGAAGAGCATCCA
GCCAGAGAATCTGGAGTACCGGATAATGCTGTCAGTTCATGGCTCCCAGCACAGTGGGATGATC
GTTAATGACACAGGACATGAAACTGATGAGAATAGAGCGAAAGTTGAGATAACGCCCAATTCA
CCGAGAGCCGAAGCCACCCTGGGGGGaTTTGGAAGCCTAGGACTTGATTGTGAACCGAGGAC
AGGCCTTGACTTTTCAGATTTGTATTACTTGACTATGAATAACAAGCACTGGTTGGTTCACAAGG
AGTGGTTCCACGACATTCCATTACCTTGGCACGCTGGGGCAGACACCGGAACTCCACACTGGA
ACAACAAAGAAGCACTGGTAGAGTTCAAGGACGCACATGCCAAgAGGCAAACTGTCGTGGTTC
TAGGGAGTCAAGAAGGAGCAGTTCACACaGCaCTTGCTGGAGCTCTGGAGGCTGAGATGGAT
GGTGCAAAGGGAAGGCTGTCCTCTGGCCACTTGAAATGTCGCCTGAAAATGGATAAACTTAGA
TTGAAGGGaGTGTCATACTCCTTGTGTACTGCAGCGTTCACATTCACCAAGATCCCaGCTGAAAC
ACTGCACGGGACAGTCACAGTGGAGGTACAGTACGCAGGGACAGATGGACCTTGCAAGGTTC
CAGCTCAGATGGCGGTGGACATGCAAACTCTGACCCCAGTTGGGAGGTTGATAACCGCTAACC
CaGTAATCACTGAAAGCACTGAGAACTCTAAGATGATGCTGGAACTTGATCCACCATTTGGaGA
CTCTTACATTGTCATAGGAGTCGGaGAGAAGAAGATCACCCACCACTGGCACAGGAGTGGCAG
CACCATTGGAAAgGCATTTGAAGCCACTGTGAGAGGTGCCAAGAGAATGGCAGTCTTGGGAG
ACACAGCCTGGGACTTTGGATCAGTTGGAGGaGCTCTCAACTCATTGGGCAAGGGCATCCATC
AAATTTTcGGAGCAGCTTTCAAATCATTGTTTGGAGGAATGTCCTGGTTCTCACAAATTCTCATT
GGAACGTTGCTGATGTGGTTGGGTCTGAACACAAAGAATGGATCTATTTCCCTTATGTGCTTGG
CCTTAGGaGGAGTGTTGATCTTCTTATCCACAGCCGTCTCTGCTTAATGATGA

Supplementary Figure 1. ZIKV-prME gene encoding sequence used for insertion in MVA-deletion site III. The cDNA sequences coding for the prM and E protein from Aminocacid 127-790 of ZIKV Yap 2007 isolate (GenBank accession no. EU545988.1YYYY) was modified by introducing silent codon alterations to remove runs of guanines or cytosines which may prevent frameshift mutations during vaccinia virus DNA replication and assure the genetic stability of the recombinant MVA genome. In addition, three poxvirus stop sequences (TTTTTNT) were inactivated to prevent premature termination of vaccinia virus-specific early transcription. To initiate transcription, an ATG was inserted just upstream of the start codon of the ZIKV-E gene. To improve transcription, Kozak-sequence was also included. The optimized ZIKV-prME gene sequence was generated by DNA synthesis (GENEWIZ, Leipzig Germany) and inserted into the MVA vector plasmids pIIIH5red-K1L to obtain the MVA expression plasmids pIIIH5red-ZIKV-prME.

Supplementary Figure 2:

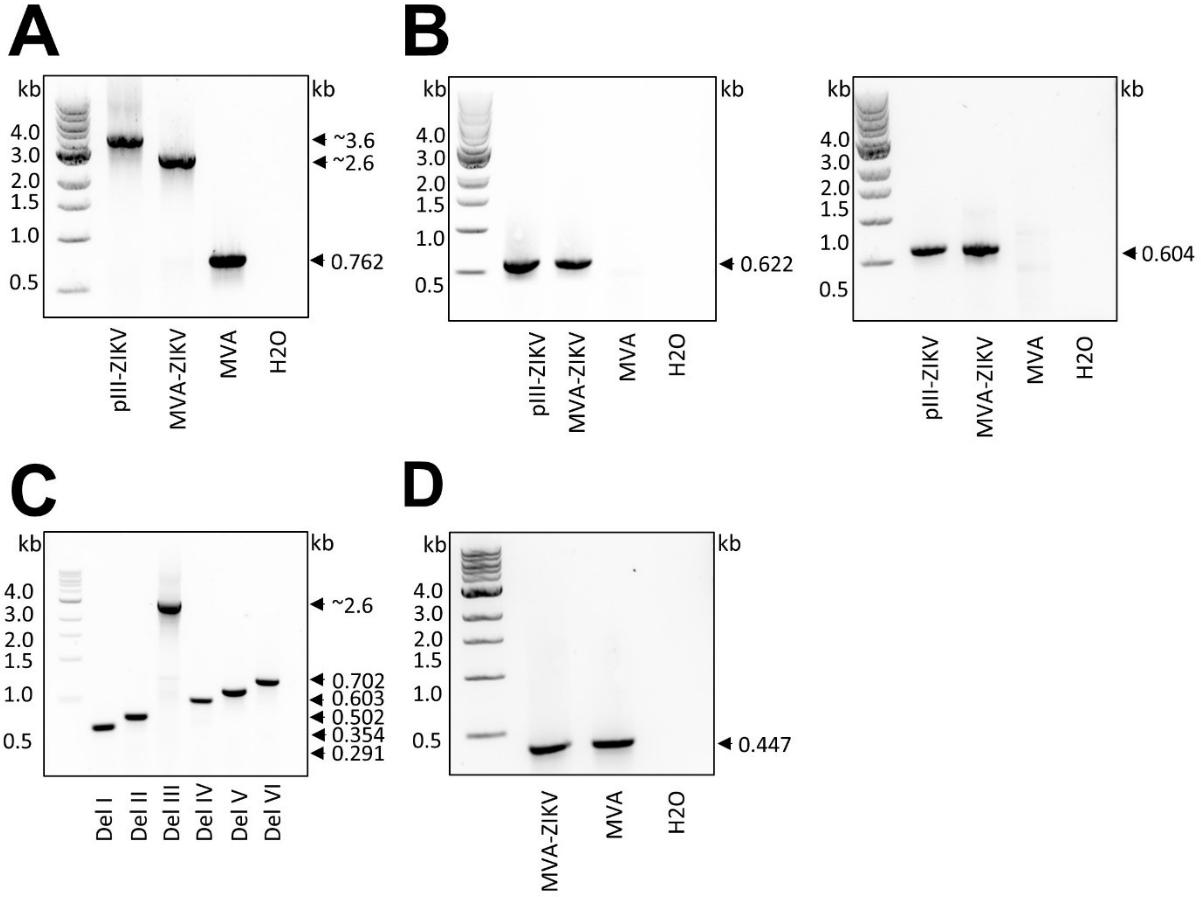

Supplementary Figure 2. Molecular analysis of the MVA-ZIKV-genome. (A) PCR analysis of genomic viral DNA confirmed stable insertion of the ZIKV-prME-genome-sequence into deletion site III of the MVA genome. The precise intragenomic deletion of the marker gene mCherry during plaque purification was revealed by amplification of a PCR product with the expected molecular weight (2.89 kb) from MVA-ZIKV-genomic DNA compared to the large product amplified from pIIIH5red-ZIKV-prME-plasmid DNA template (3.65 kb). The deletion III site-specific oligonucleotide primers amplified a characteristic 0.762 kb DNA fragment from genomic, non-recombinant MVA DNA. (B) PCR analysis of genomic viral DNA to monitor sequences of the ZIKV-prME-gene in the MVA-ZIKV-genome. Two different PCRs were used to assess the integrity of the ZIKV-prME-gene sequence inserted in the MVA-genome. Specifically amplified DNA fragments demonstrated the expected molecular weight with 0.5 kp (specific for ZIKV-prME nucleotides 53-1443) and 0.714 kb (specific for ZIKV-prME). (C) PCR analysis demonstrated the genetic stability for six loci in the

MVA-ZIKV-genome (deletion sites Del I-VI) including the heterologous ZIKV-prME-gene sequences inserted into the site of deletion III (Del III) with the amplification of characteristic size DNA fragments from viral DNA. (D) PCR analysis of genomic viral DNA to monitor sequences of the C7L gene locus. PCR amplification of a specific 0.447 kb DNA fragment from the MVA C7L gene sequence suggested the integrity of the C7L gene locus in the recombinant MVA-genome in comparison to the non-recombinant MVA genome (MVA). The C7L gene is non-essential for MVA growth in chicken fibroblast cultures but the gene function is necessary to maintain unimpaired expression of MVA or recombinant genes under transcriptional control of vaccinia virus-specific late promoters.

Supplementary Figure 3:

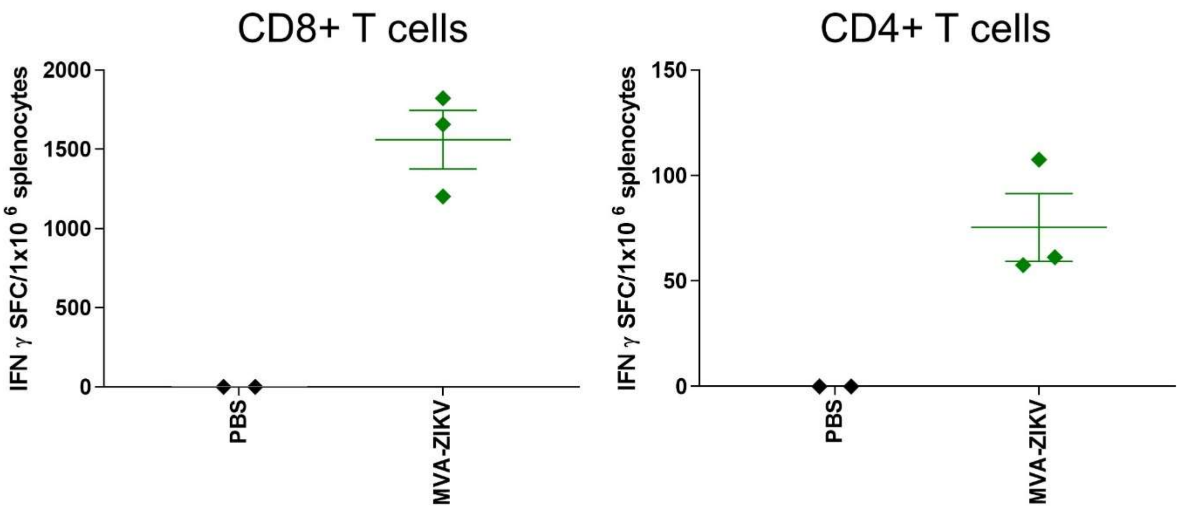

Supplementary Figure 3: ZIKV-specific memory T cells induced after MVA-ZIKV vaccination in IFNAR-/- mice without ZIKV challenge infection. Mice were PBS-mock vaccinated (n=2) or vaccinated with a standard dosage of $1x10^8$ PFU MVA (n=3) using the intramuscular route, and analyzed for IFN-γ SFC after stimulation with $E_{294-302}$ peptide (CD8+ T cells) or $E_{646-664}$ peptide (CD4+ T cells) measured by ELISPOT assay 6 months after initial vaccination. Each dot indicates for a single mouse. The horizontal lines and associated error bars indicate for mean ± standard error of the mean (SEM). Differences between the groups were analyzed by Mann-Whitney Test. Asterisks represent statistically significant differences between two groups: * $p < 0.05$.

Supplementary Figure 4:

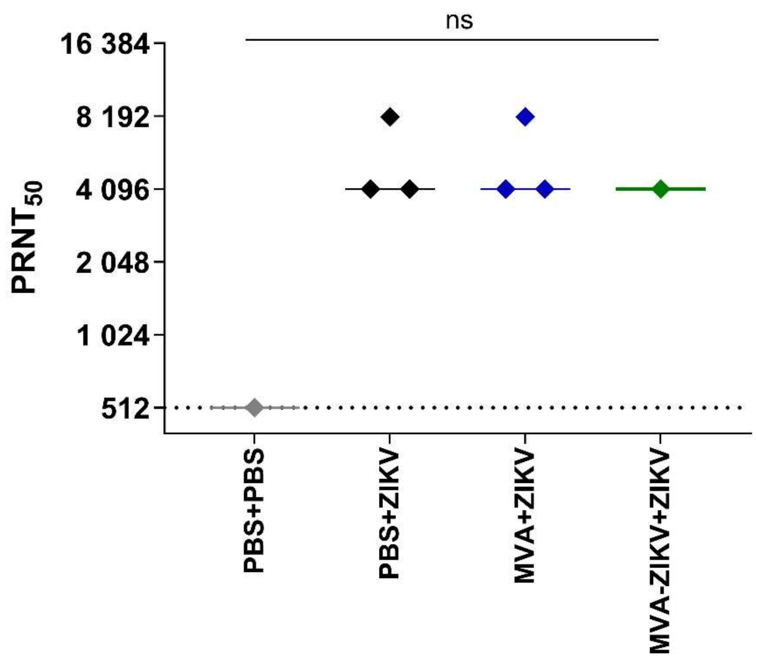

Supplementary Figure 4. ZIKV-neutralizing antibodies in IFNAR-/- mice after MVA-ZIKV vaccination and ZIKV challenge 28 days later: IFNAR-/- mice were challenged with ZIKV 28-days after intramuscular vaccination with MVA-ZIKV (10^8 PFU, n=5). Empty MVA (n=4) or PBS-vaccinated mice (mock-vaccinated animals, n=3) were used as vaccination controls. PBS-vaccinated and PBS-challenged mice (mock-mock animals, n=4) were used as infection controls. Sera were prepared at the day of death (PBS+ZIKV, MVA+ZIKV: 10-11 dpc, PBS+PBS, MVA-ZIKV+ZIKV: 28 dpc) and analyzed for ZIKV-neutralizing antibodies by plaque reduction assays (PRNT50).

Supplementary Figure 5:

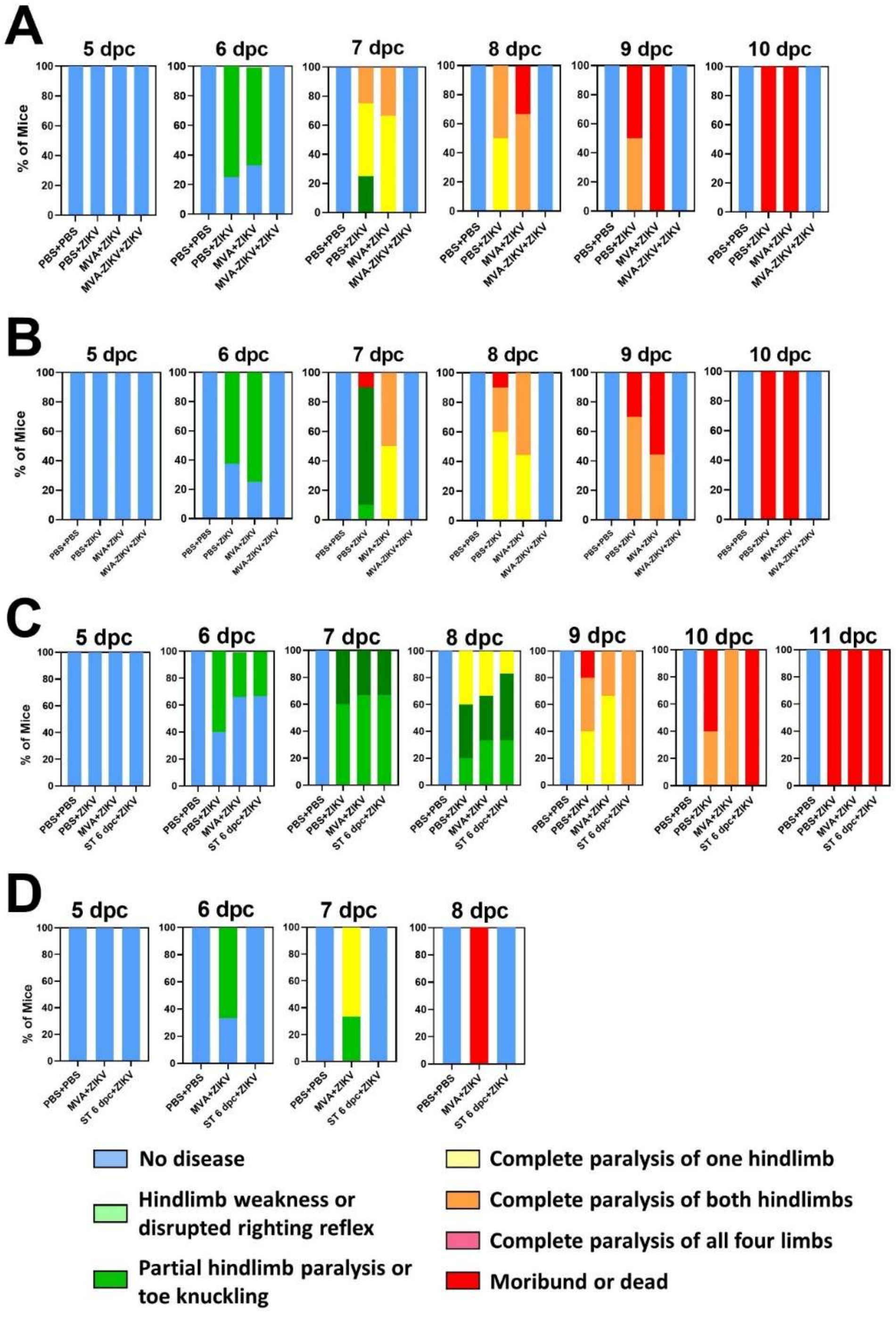

Supplementary Figure 5: Clinical disease after ZIKV challenge infection in IFNAR-/-mice. Development of ZIKV-specific clinical disease was monitored daily after ZIKV challenge infection using a clinical score sheet including the different categories of ZIKV-specific disease: (blue) no disease, (light green) hindlimb weakness or disrupted righting reflex, (green) partial hindlimb paralysis or toe knuckling, (yellow) complete paralysis of one hindlimb, complete paralysis of both hindlimbs,

(orange) complete paralysis of all four limbs, or (red) moribund or dead; mice with a disease score of complete paralysis of two or more hindlimbs or moribund as measured by weight loss of more than 20% of initial body weight loss were euthanized. (A) Clinical disease change was monitored daily for IFNAR-/- mice that received MVA-ZIKV-vaccination 28 days before ZIKV challenge infection. (B) Clinical disease change was monitored daily for IFNAR-/- mice that received MVA-ZIKV-vaccination 2 days before ZIKV challenge infection. (C, D) The protective capacity of passively transferred serum from different vaccination schedules has been tested against a lethal ZIKV challenge infection 24 hours after serum transfer. (C) Clinical disease change was monitored daily for IFNAR-/- mice that received sera from IFNAR-/-mice that had been vaccinated with empty MVA-vector control or MVA-ZIKV 2 days before ZIKV challenge infection (sera: 6 days post ZIKV challenge). (D) Clinical disease change was monitored daily for IFNAR-/- mice that received sera from IFNAR-/- mice that had been vaccinated with empty MVA-vector control or MVA-ZIKV 28 days before ZIKV challenge infection (sera: 6 days post ZIKV challenge).

Supplementary Figure 6:

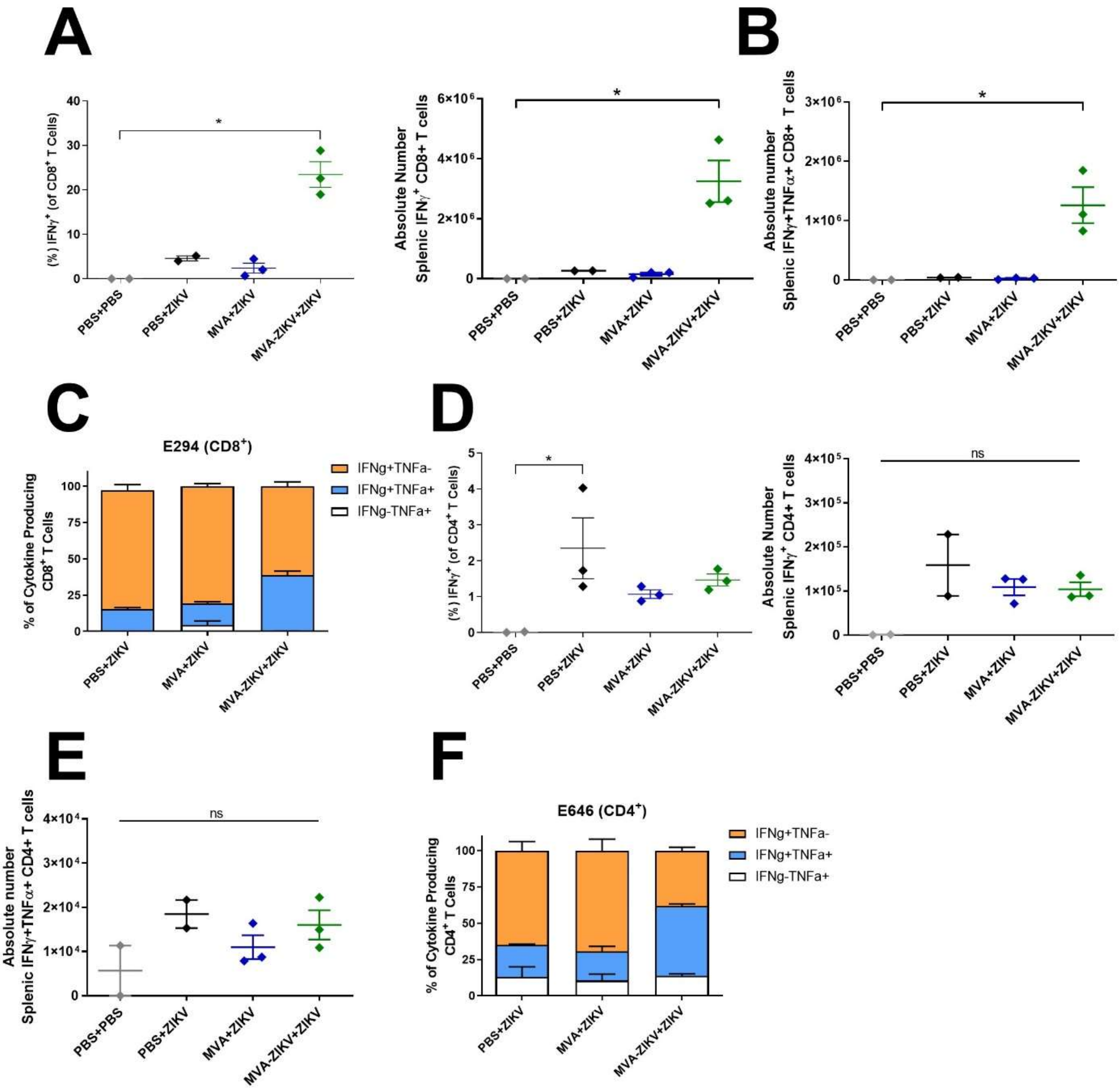

Supplementary Figure 6. Activation of ZIKV-specific T cells. Groups of IFNAR-/- mice (n = 2 - 4) were vaccinated with 10^8 PFU of MVA-ZIKV and challenged with ZIKV 2 days later. MVA and mock (PBS) vaccinated plus challenged mice were used as controls. Splenocytes were collected and prepared at day 6 post-challenge. Splenocytes were stimulated with the MHC class I-restricted peptide $E_{294-302}$ (IGVSNRDFV) (A-C) and the MHC class II-restricted peptide $E_{646-664}$ (GRLITANPVITESTE) (D-F) derived from the ZIKV-prME protein and tested by IFN-γ / TNF-α ICS and flow cytometric analysis. Graphs show the frequency and absolute number of IFN-γ+ CD8+ T cells (A) and CD4+ T cells (D). Absolute number of IFN-γ+TNF-α+ CD8+ T cells (B) and CD4+ T cells (E). Frequency of cytokine producing CD8+ T cells (C) and CD4+ T cells (F) with combinations

of IFN-γ and TNF-α. Each dot indicates for a single mouse. The horizontal lines and associated error bars indicate for mean ± standard error of the mean (SEM). Data were analysed by Kruskal-Wallis Test. Asterisks represent statistically significant differences between two groups. ns = non-significant, * $p < 0.05$.

Supplementary Figure 7:

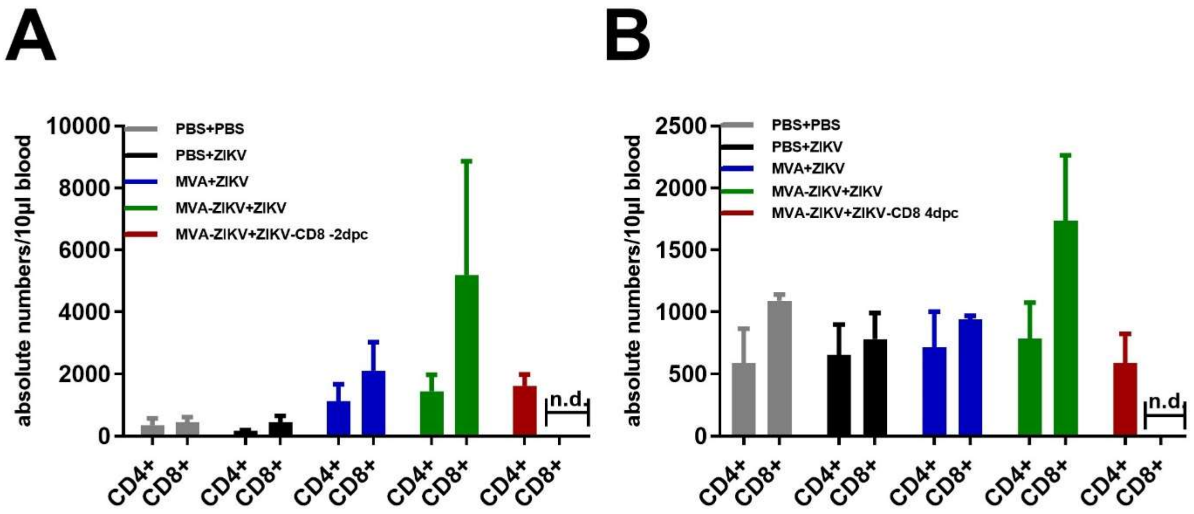

Supplementary Figure 7. Depletion of CD8+ T cell as confirmed by flow cytometry. To confirm the successful depletion of CD8+ T cells, blood samples were taken from IFNAR-/- mice treated with anti-CD8 antibody at day -2 or 4 post-challenge (dpc). Cells were stained with anti-mouse CD4 and CD8 antibodies and analyzed by flow cytometry. Graphs show the absolute number of CD8+ and CD4+ T cells in the blood of non-depleted and CD8+ T cell depleted IFNAR-/- mice at day -2 dpc (A) and day 4 dpc (B). The error bars indicate for mean ± standard error of the mean (SEM).

Supplementary Figure 8:

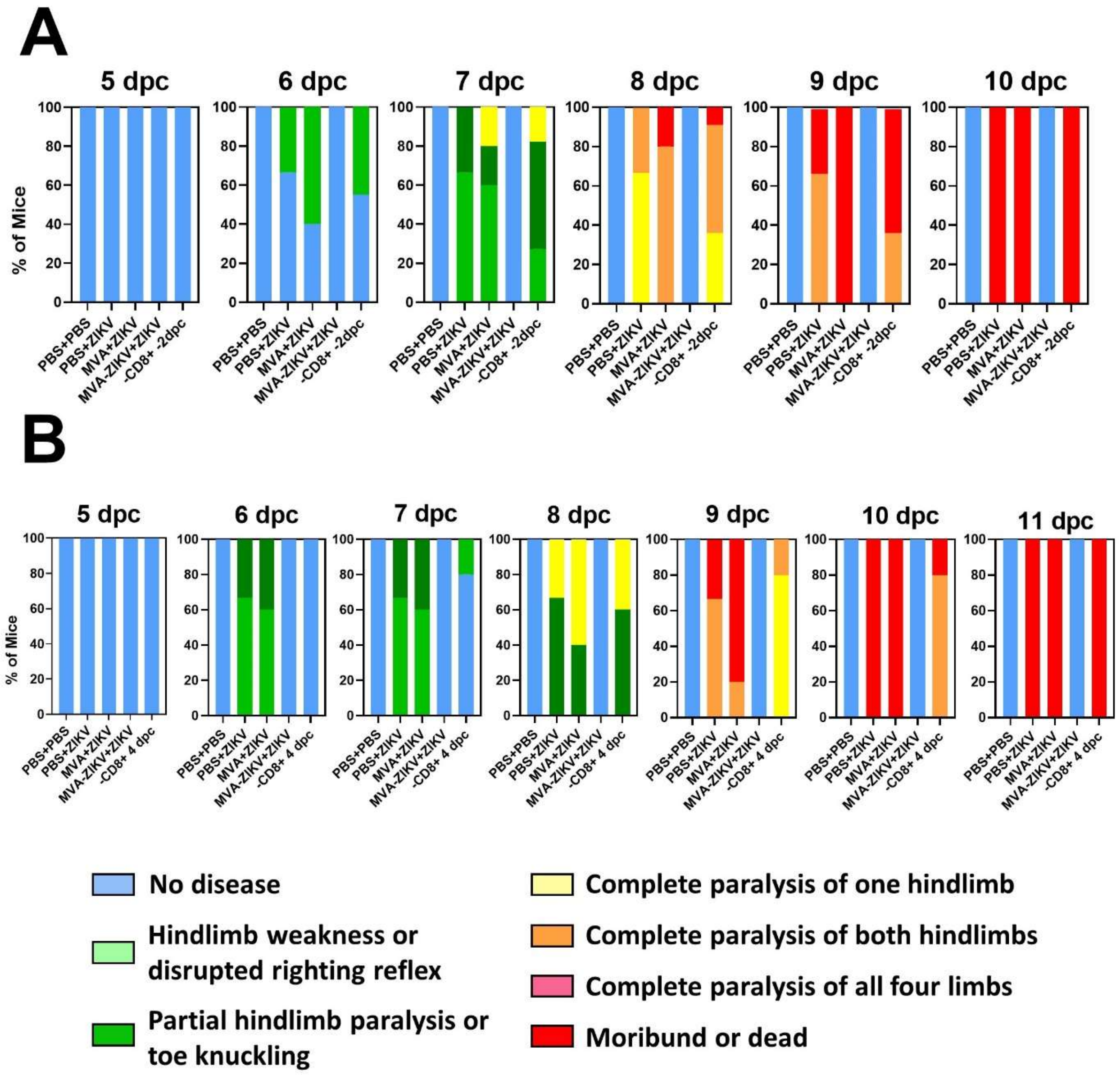

Supplementary Figure 8: Clinical disease after ZIKV challenge infection in MVA-ZIKV-vaccinated and CD8+ T cell depleted IFNAR-/-mice. Development of ZIKV-specific clinical disease was monitored daily after ZIKV challenge infection using a clinical score sheet including the different categories of ZIKV-specific disease: (blue) no disease, (light green) hindlimb weakness or disrupted righting reflex, (green) partial hindlimb paralysis or toe knuckling, (yellow) complete paralysis of one hindlimb, complete paralysis of both hindlimbs, (orange) complete paralysis of all four limbs, or (red) moribund or dead; mice with a disease score of complete paralysis of two or more hindlimbs or moribund as measured by weight loss of more than 20% of initial body weight loss were euthanized. (A-B) Clinical disease change was monitored daily in IFNAR-/- mice depleted of CD8+ T

cells at different time points after vaccination (-2 dpc, 4 dpc) and ZIKV challenge 2 days after intramuscular vaccination with MVA-ZIKV; 10^8 PFU). MVA-ZIKV (n=5) vaccinated animals were compared to MVA (n=5) or mock (PBS; n=3) vaccinated control animals. PBS-vaccinated + PBS-challenged mice (mock-mock, n=4) were used as negative controls. (A) Clinical disease was monitored daily for IFNAR-/- mice that had been depleted for CD8+ T cells at -2 dpc or (B) IFNAR-/- mice that had been depleted for CD8+ T cells at 4 dpc.

Supplementary Figure 9:

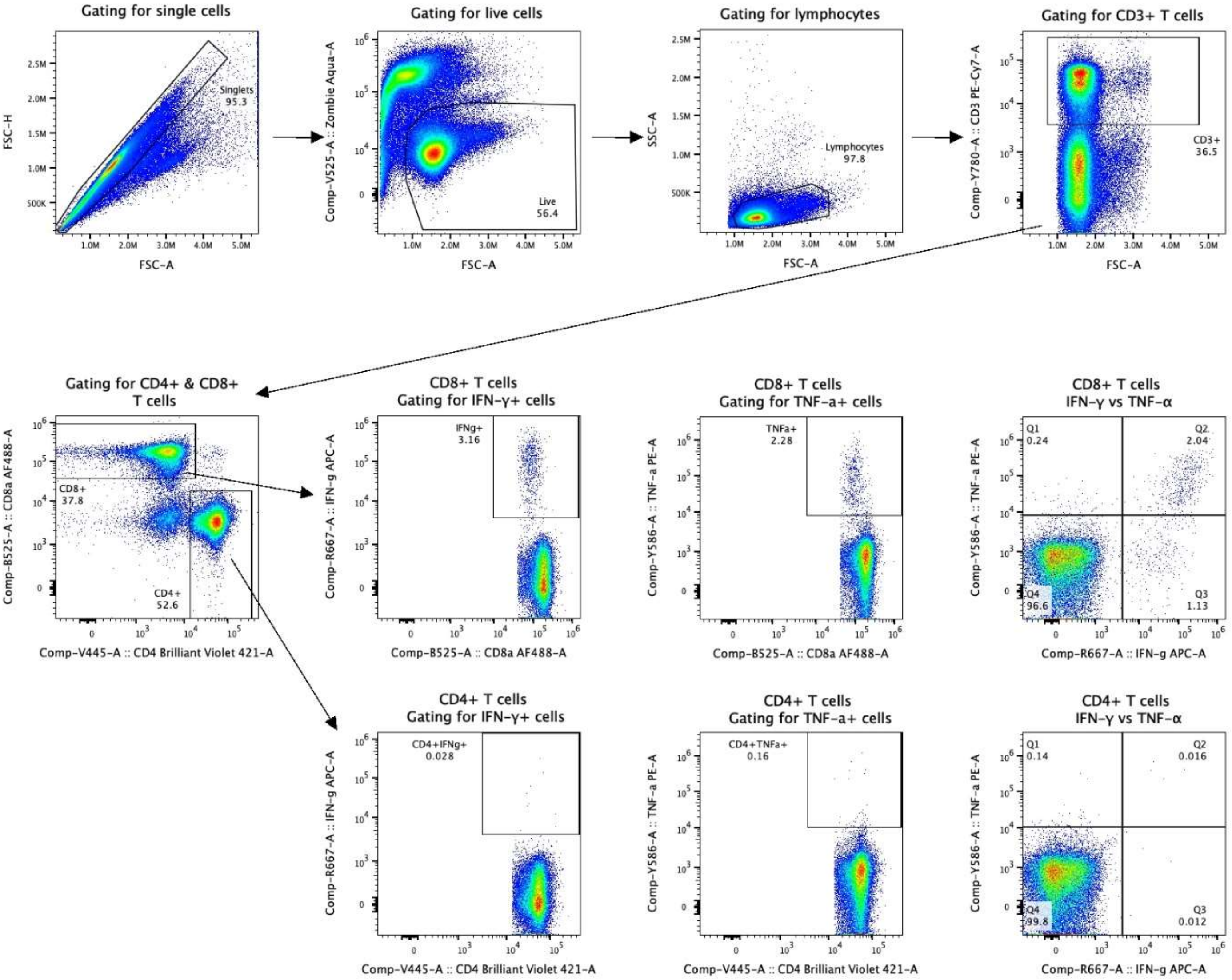

Supplementary Figure 9: Gating strategy used for ICS

To analyze for cytokine producing cells after stimulation with ZIKV- or MVA-specific peptides, single cells were selected by gating for Forward Scatter Area (FSC-A) versus FSC height (H). Dead cells were then excluded by gating FSC-A versus the viability dye Zombie Aqua, and then lymphocytes were selected by gating FSC-A against Side Scatter Area (SSC-A). CD3+ T cells were selected by gating FSC-A versus CD3, which was followed by the gating of CD4+ and CD8+ T cells using CD8 versus CD4 flow cytometry plots. Next, IFN-γ+, TNF-α+ cells and dual cytokine producing cells were gated in each T cell population as shown.

Original and unprocessed scans of blots and microscopy images

Supplementary Figure 10:

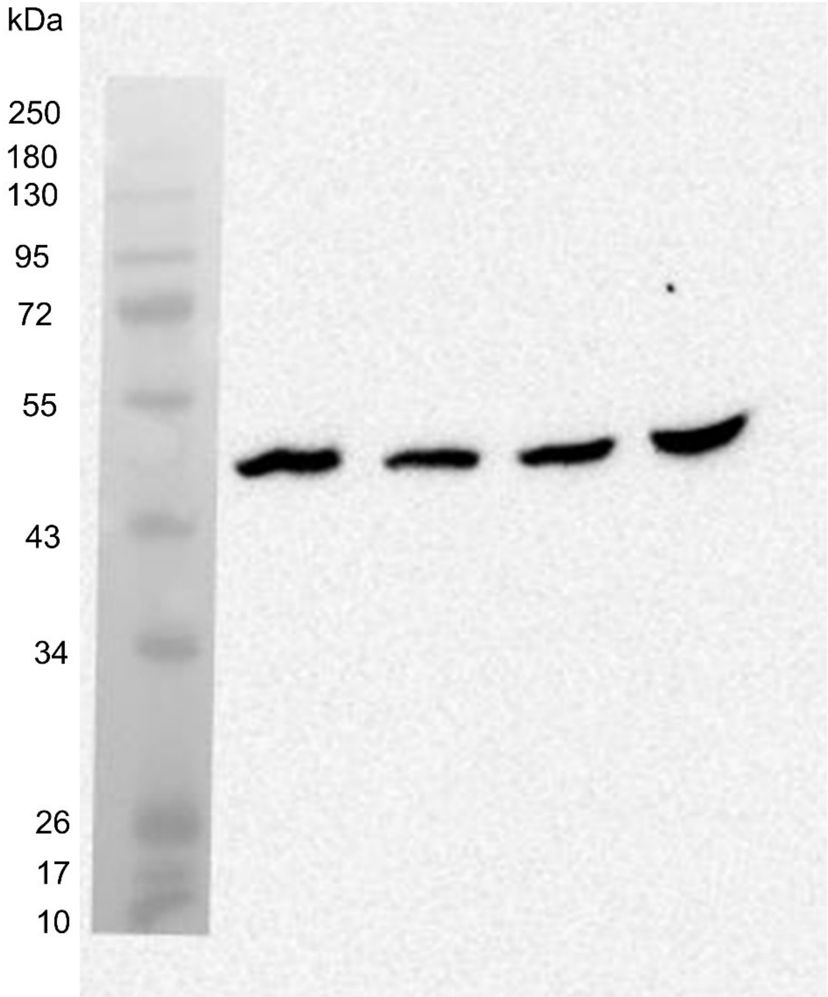

Supplementary Figure 10: Full unedited blot from Fig. 1C. Synthesis and processing of ZIKV-E-protein in MVA-ZIKV-E. Blot shown is uncropped.

Supplementary Figure 11:

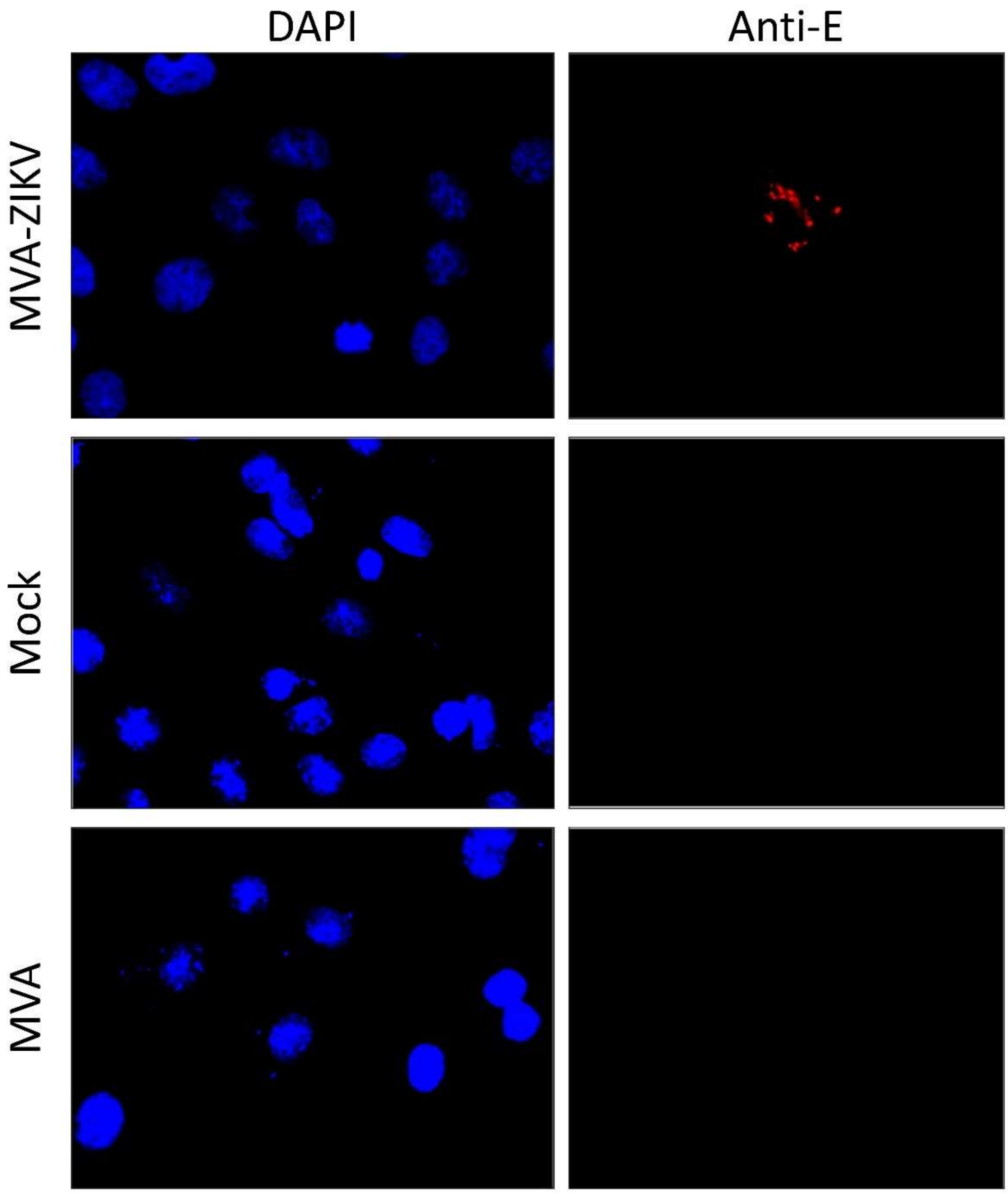

Supplementary Figure 11: Full unprocessed scans of microscopy images from Fig. 1E. In situ immunofluorescence of ZIKV-E protein in MVA, mock, or MVA-ZIKV-infected Vero cells. Images shown are uncropped.

Supplementary Figure 12

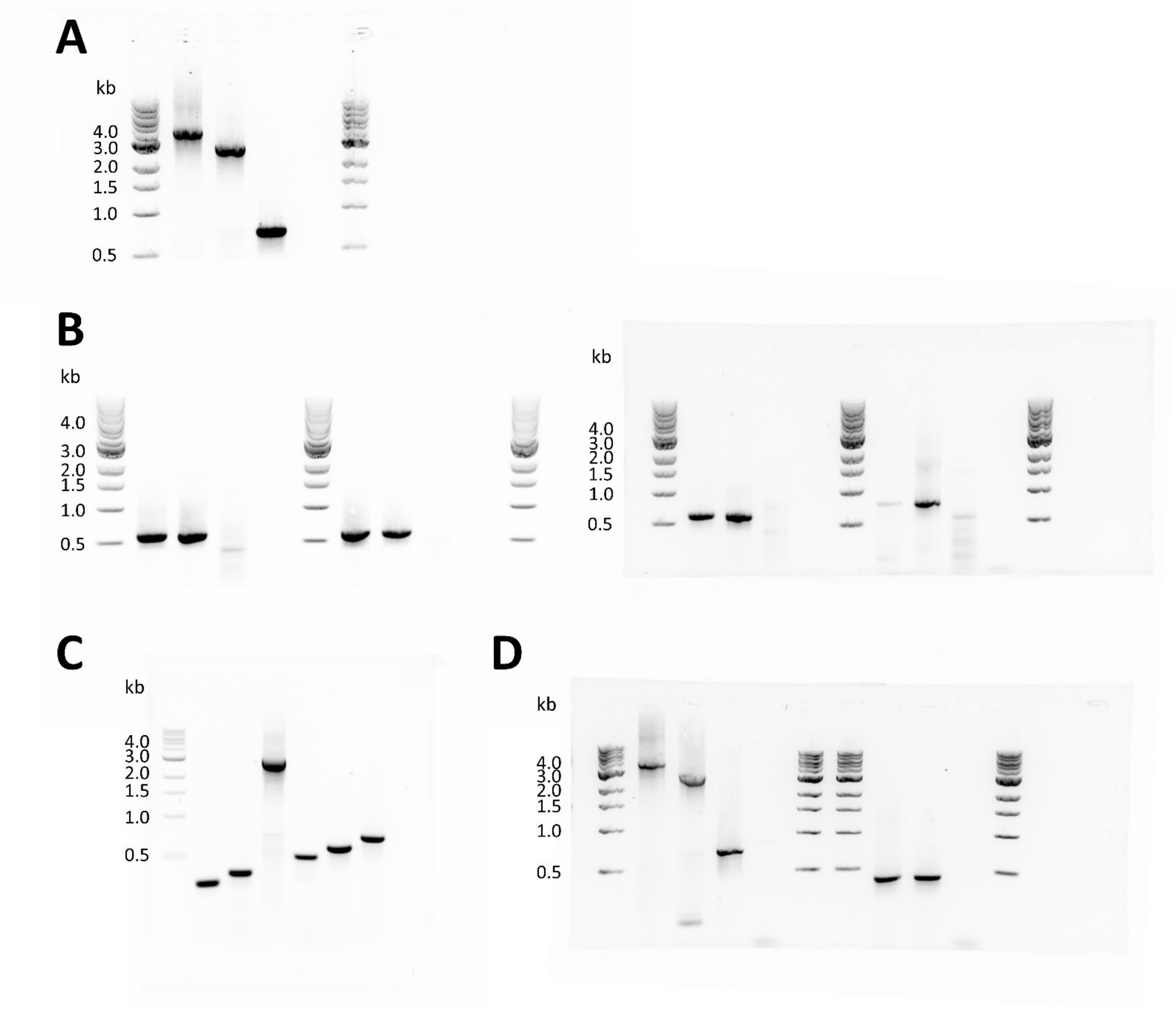

Supplementary Figure 12: Full unedited blots from Supplementary Figure 2: Molecular analysis of the MVA-ZIKV-genome. Blots shown are uncropped.
